# Unexpected Pathway in Organic Semiconductor Nanoparticle Formation

**DOI:** 10.1021/acsnano.5c07335

**Published:** 2025-07-31

**Authors:** Arthur E. Bouchez, Connor R. Firth, Arnau Bertran, Colin Jeanguenat, Jun-Ho Yum, Kevin Sivula

**Affiliations:** Laboratory for Molecular Engineering of Optoelectronic Nanomaterials, Institute of Chemical Sciences and Engineering, 27218 École Polytechnique Fédérale de Lausanne (EPFL), Lausanne CH-1015, Switzerland

**Keywords:** photocatalysis, hydrogen, water reduction, emulsion, size control

## Abstract

Organic semiconductor (OSC) nanoparticles (NPs) are promising for numerous applications including greener organic photovoltaics and heterogeneous photocatalysts for solar H_2_ production. Single component or mixed bulk-heterojunction (BHJ) OSC NPs are commonly prepared from conventional polymer OSCs via the miniemulsion-evaporation method using ultrasonication. However, realizing the expected NP size control with this approach remains elusive, limiting optimization. Here, we demonstrate that the presumed miniemulsion-evaporation mechanism is not the principal pathway forming NPs. Predominantly, a direct extraction of OSCs from the organic to the aqueous phase during ultrasonication results in NP formation prior to organic solvent evaporation, rendering NP size insensitive to emulsion parameters. By replacing ultrasonication with lower-energy shear mixing, we control the competition between these pathways, achieving tunable NP sizes via a true emulsion-evaporation mechanism. This enables the first demonstration of BHJ NP size effects on photocatalytic H_2_ evolution, with a ∼2-fold increase in H_2_ production when reducing NP diameter from 230 to 160 nm. However, the observed ∼14-fold higher performance of direct-extraction BHJ NPs (25 nm diameter) highlights the need to reassess OSC NP formation. Overall, this work advances an understanding of photocatalytic activity via size optimization and offers a greener processing route by eliminating organic solvent evaporation.

## Graphical abstract

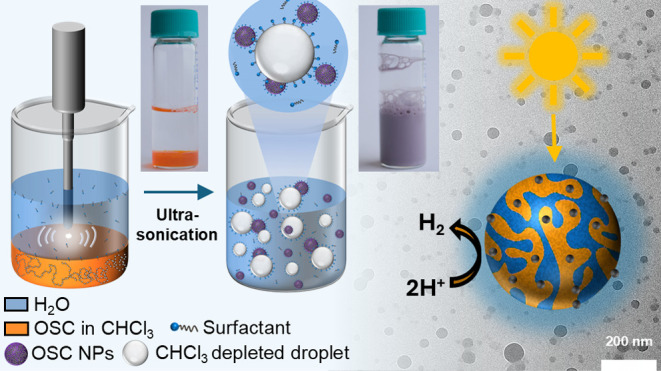

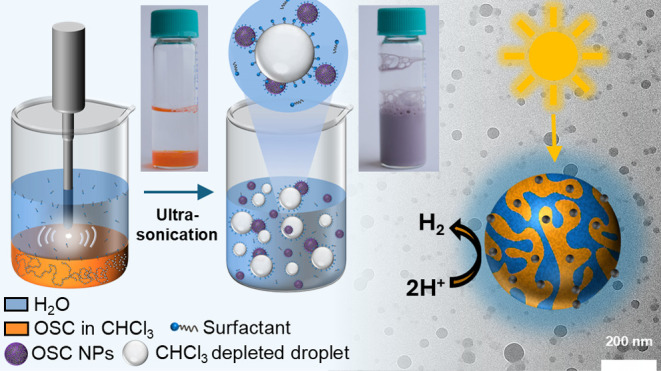

## Introduction

Nanoparticles (NPs) prepared from polymer or small molecule π-conjugated organic semiconductors^1,2^ (OSCs) are under intense investigation as promising materials for the green processing of organic photovoltaics and transistors,^3,4^ phototherapy,^5^ sensing and imaging,^6^ and as heterogeneous photocatalysts for solar to fuel conversion.^7^ Considering photocatalysis in particular, producing hydrogen (H_2_) from sunlight and water will likely become an integral process for renewable energy storage and chemical feedstock supply in a future sustainable energy economy.^8^ Heterogeneous photocatalysis is projected to be one of the most cost-effective ways of producing green H_2_, due to the simplicity and scalability of producing and applying nanoparticle (NP) photocatalyst dispersions.^9–11^ However, identifying photocatalyst systems that can satisfy the requirements of high efficiency, long-term stability, and scalability at the global level remains a significant challenge. Recently, OSC NPs composed of electron-donating (D) and electron-accepting (A) materials forming a bulk-heterojunction (BHJ) have demonstrated impressive performance compared to conventional inorganic photocatalyst materials.^1,12,13^ While BHJ NPs offer appealing earth abundance, processability, and molecular tuneability, research into their operation as photocatalysts remains in a preliminary stage. Fundamental advances in understanding and controlling BHJ NP formation are still needed in order to optimize key parameters like D/A demixing and NP size. Indeed, despite the obvious importance of particle size on surface area, light absorption and photogenerated charge extraction, controlling this aspect remains a challenge.

The ability to control NP size certainly depends on the preparation route. BHJ or single component OSC NPs are typically formed via the self-assembly of conventional polymer or small molecule OSCs from solution^4,14^ using a nanoprecipitation^15–17^ or miniemulsion-evaporation^7,18,19^ method. The miniemulsion method is particularly promising toward controlling NP formation as size regulation with this method is well documented using standard polymers like poly­(methyl methacrylate).^20^ Briefly, an aqueous solution of surfactant is emulsified by ultrasonication with an immiscible volatile organic solvent containing the dissolved OSCs. The resulting miniemulsion, which has the organic solvent as the dispersed phase (with droplet diameters in the 20–500 nm range^21^), is subsequently heated to evaporate the solvent, concentrating and solidifying the OSCs to yield an aqueous dispersion of NPs stabilized by the surfactant. Simply changing the initial OSC concentration or emulsion droplet size should regulate the average NP size as demonstrated with a number of nonsemiconducting polymers.^20,22–26^ However, despite the large number of published systems using different OSC NP formation parameters (Table S1),^4,27^ the control of NP size through this ultrasonication miniemulsion-evaporation route has not been convincingly demonstrated with organic semiconductors. Most reports demonstrate broad size distributions with NPs diameters ranging between 30 and 150 nm, despite using different OSCs and surfactants spanning concentration ranges of 2 orders of magnitude. While some reports claim a significant size control, typically only dynamic light scattering (DLS) measurements were given, and considering the known issues of DLS in characterizing organic NP suspensions with wide dispersity and low scattering intensity,^28–31^ the accuracy of the reported size remains questionable. This lack of convincing ability to control size hinders photocatalyst optimization and represents a major roadblock in the field. Moreover, the discrepancy between the expected size control and experimental observations raises fundamental questions about the NP formation mechanism.

Here we show that the conventional miniemulsion-evaporation mechanism is not the predominant pathway leading to the formation of BHJ or single-component OSC NPs, unlike previously considered. Our study reveals that the vast majority of NPs are formed via direct extraction of the OSC from the organic phase into the aqueous phase during ultrasonication and prior to organic solvent evaporation. Using a combination of direct particle size measurements from electron microscopy and a DLS measurement protocol verified with standard polymer NP samples, we demonstrate that the size of the OSC NPs formed through this alternative pathway is insensitive to miniemulsion parameters, explaining previous experimental observations. By using lower-energy shear mixing emulsification instead of ultrasonication, we establish control over the yields of these two competing pathways and demonstrate the size tuneability of NPs formed through a true miniemulsion-evaporation mechanism. Following this approach, we show that smaller particle sizes yield higher photocatalytic H_2_ evolution rates within the studied size range.

## Results and Discussion

Given the importance of controlling the NP size in the field of heterogeneous photocatalysis and the relative complexity of state-of-the-art BHJ NP systems, we first sought to understand the influence of various process parameters on the resulting NP size using a model system. The polymer OSC poly­(3-hexylthiophene) (P3HT, see structure Figure S1) was chosen for initial NP formation investigations owing to its color change from orange, when dissolved in a good solvent (e.g., CHCl_3_), to purple, when aggregated in a bad solvent (e.g., water) or as a solid, due to π–π stacking.^32^ Following a miniemulsion-evaporation protocol similar to those previously described in the literature^7,27,33,34^ and understood by the authors to operate in the traditional mechanism shown schematically in Figure 1a (top), we used sodium dodecyl sulfate (SDS) as surfactant and P3HT at various concentrations in CHCl_3_. Immediately after emulsification of this “P3HT@SDS” system using ultrasonication the formation of CHCl_3_ droplets in water (size ∼1–10 μm by optical microscopy, see Figure S2) were observed. However, the obvious color change from the initial orange solution to a purple emulsion indicated aggregation of P3HT during ultrasonication (as shown for a P3HT concentration of 1 mg/mL in photographs of the sample vials “Before” and “After” in Figure 1b, as well as the UV–vis spectra Figure S3). Allowing 24 h for the emulsion to rest, the CHCl_3_ droplets settled at the bottom of the vial, revealing the top aqueous phase to be dark purple (see “After 24 h” photograph in Figure 1b), and suggesting that the P3HT was no longer dissolved in the CHCl_3_ phase. This behavior was consistent for a wide range of P3HT concentrations (0.1–10 mg/mL, see photographs in Figure S4), and even in the absence of surfactant (Figure S5). Electron microscopy imaging of the dried aqueous phase revealed the presence of P3HT NPs with diameters primarily in the 10–60 nm range (Figure S6). We note that these observations are not compatible with the classic miniemulsion-evaporation mechanism where CHCl_3_ evaporation is required to form the OSC NPs, but rather consistent with an alternative mechanism where the OSC is directly extracted during the ultrasonication step (as shown schematically in Figure 1a, bottom).

**Figure 1:**
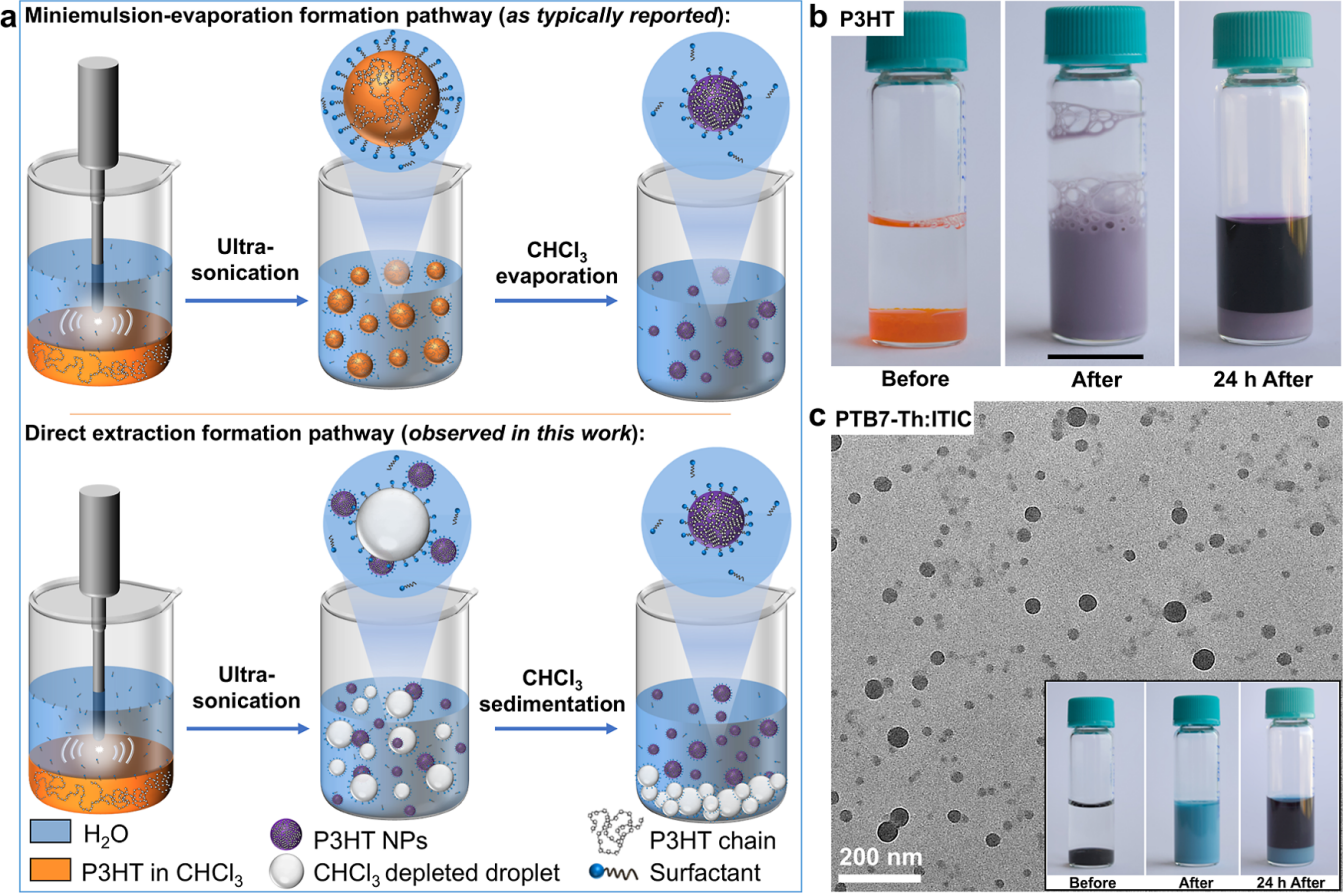
(a) Schematic of the conventional miniemulsion-evaporation formation pathway using ultrasonication, as described in the literature (top) and of the direct extraction pathway observed in this work (bottom). (b) Photographs of the P3HT@SDS system (1 mg/mL P3HT, 5 mg/mL SDS) at different points during the preparation using ultrasonication: before (left), immediately after (center) and 24 h after (right) ultrasonication. No heating was applied to remove the CHCl_3_. The scale bar is 16 mm. (c) TEM image of the PTB7-Th:ITIC(3:7)@TEBS NPs (1 mg/mL PTB7-Th:ITIC, 5 mg/mL TEBS) obtained in the aqueous phase by ultrasonication after settling of the CHCl_3_. Inset: pictures of the PTB7-Th:ITIC@TEBS system during the preparation at the same points as in panel b.

To show that this behavior is not limited to P3HT, and to more rigorously quantify the NP size dependence on emulsion conditions, we shift our attention to the more application-relevant OSC blend of PTB7-Th:ITIC (mixed at a 3:7 ratio by mass, see chemical structures in Figure S1), and the surfactant sodium (3-thienyl)­ethyloxy-4-butylsulfonate (TEBS). This “PTB7-Th:ITIC@TEBS” system was chosen as it has been previously reported for the preparation of high performance BHJ NPs for photocatalytic H_2_ evolution.^35^ Similar to the P3HT and consistent with the “direct extraction” mechanism, upon ultrasonication to form an emulsion of CHCl_3_ droplets in water (see Figure S7) the aqueous phase turned blue, which is clearly seen after sedimentation of the CHCl_3_ (see photographs inset in Figures 1c and S8) for a wide range of OSC concentrations from 0.1 to 10 mg/mL. NPs with diameters primarily in the 10–60 nm range were observed in the aqueous phase as confirmed by transmission electron microscopy, TEM (Figures 1c, S8 and S9). It should be noted that for PTB7-Th, ITIC, and other state-of-the-art OSCs, there is little or no color change upon aggregation/solidification (Figures S3 and S8), like that observed in P3HT, making the direct extraction phenomenon difficult to observe unless the CHCl_3_ droplets are allowed to sediment over several hours. Indeed, in typically reported miniemulsion-evaporation protocols, CHCl_3_ evaporation is performed immediately after ultrasonication in order to avoid droplet coalescence. This appears to be a plausible explanation for why the direct extraction phenomenon has not been reported before.

Quantification of the PTB7-Th:ITIC@TEBS NP diameter and size dispersity by directly measuring >1000 particles from TEM images (in order to avoid errors in DLS measurement) suggests no significant dependence of the NP diameter, *d*, on the OSC concentration in the initial CHCl_3_ solution, *C*, as shown in the violin plots of the distribution of *d* for *C* = 0.1, 1, 5, and 10 mg/mL in Figure 2a (see examples of TEM images in Figure S9). In each case the median diameter was around 25–30 nm with 50% of the particles between *d* = 20 and 40 nm and the distribution thinned out by 100–150 nm. According to the classic miniemulsion-evaporation mechanism, the average *d* should be proportional to *C* ^1/3^ (see Methods section for a full explanation).^36^ However, plotting the median *d* with respect to *C* on a log–log scale revealed a clear deviation of the NP size from the expected cube root trend (Figure 2b). We note that comparison of the NP diameter measured by DLS for the same samples shown in Figure 2a gave similar results with no accordance with the expected cube root trend (Figure S10), although the particle size was overestimated compared to the direct TEM measurements. Indeed, comparing the measured diameter and size dispersity of standard polystyrene NPs using TEM and DLS reveals that DLS tends to overestimate sizes (see Figure S11). This overestimation makes it less reliable for accurately determining true NP sizes, particularly in the 20–50 nm range, and only suitable for comparing different NP batches. Moreover, the discrepancy between DLS and TEM measurements suggests that previous studies (as outlined in Table S1) may have overestimated the OSC NP size.

**Figure 2:**
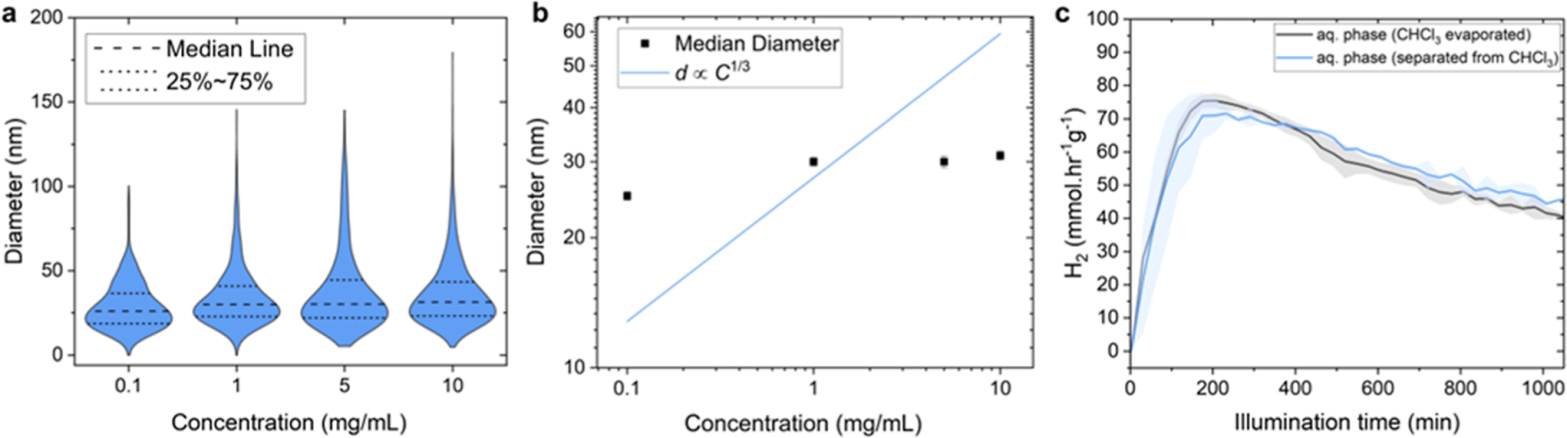
(a) Violin plots of the NP size distribution of the PTB7-Th:ITIC(3:7)@TEBS NPs from the aqueous phase (after ultrasonication and sedimentation of CHCl_3_) as measured by TEM for different concentration of OSCs in the CHCl_3_ and all other parameters the same. The number of particles of a given diameter is represented by the width of the distribution. (b) Logarithmic plot of the median NP diameter vs OSC concentration for the samples in panel a. The blue line represents the best-fit curve with a slope of 1/3 (expected for the miniemulsion-evaporation pathway). The error bars indicate the median standard error in the NP diameters. (c) H_2_ evolution rates under simulated 1 Sun illumination for the PTB7-Th:ITIC(3:7)@TEBS NPs obtained from the preparation at 5 mg/mL of OSC (nominal Pt loading = 16 wt %, ascorbic acid concentration = 0.23 M) after evaporating the CHCl_3_ (black trace) or separating it by sedimentation (blue trace). The curves are an average of measurements performed with three different samples; the shaded area represents the standard deviation. TEBS concentration was 5 mg/mL for all samples.

Besides OSC concentration, varying other emulsification parameters such as surfactant concentration (2.5–50 mg/mL TEBS) and ultrasonication time (1 s–30 min) and power (1–40% amplitude),^4,23,25,37^ also did not yield significant changes in NP size in comparison to the changes of parameters and TEBS concentration. Indeed, median *d* were found to vary less than 25% compared to a standard sample prepared with 5 mg/mL TEBS, 1 min ultrasonication at 40% amplitude (as measured by DLS), and no trends in NP size were evident (Figure S12). Moreover, the NP diameter appeared to be insensitive to the organic solvent evaporation step usually performed during miniemulsion NP preparation protocols. Indeed, either evaporating the CHCl_3_ directly after ultrasonication or allowing the CHCl_3_ to settle and then removing the aqueous phase gave particle dispersions with similar diameters (Figure S13). This is likely due to the almost complete extraction of the OSCs from the organic phase to the aqueous phase after even short ultrasonication times, as illustrated by estimating the weight fraction of OSC remaining in the CHCl_3_ after ultrasonication and separation of the organic phase (Figure S14). Despite varying the initial OSC concentration from 0.1 to 10 mg/mL, consistently less than 1 wt % of the OSC remained in the organic phase, as measured by UV–vis spectroscopy.

The CHCl_3_ evaporation step also did not affect the photocatalytic performance of the resulting BHJ NPs. Photocatalytic H_2_ evolution, measured at atmospheric pressure in the purge gas configuration (see schematic Figure S15)^35^ with in situ Pt cocatalyst photodeposition and ascorbic acid as a hole scavenger, gave peak rates of ∼70 mmol H_2_ hr^–1^ g^–1^ (which is comparable to similar reported systems^12,27,38^ ) regardless of whether CHCl_3_ was evaporated or separated by sedimentation, as shown in the plots of H_2_ evolution rate as a function of illumination time in Figure 2c. The shape of these H_2_ evolution rate curves are typical for this system and were described in recent work.^35^

Overall, the results with the P3HT@SDS and PTB7-Th:ITIC@TEBS systems support our conclusion that the formation of OSC NPs by ultrasonication of a CHCl_3_:water mixture does not follow the accepted mechanism for the miniemulsion-evaporation method.^4,20,22–26^ Given the wide parameter range tested and the similarity to previously reported NP formation protocols, it is reasonable to conclude that this discrepancy must have been the case for earlier disclosed ultrasonication miniemulsion-evaporation studies using various OSC NP systems. Thus, our observations reveal a general misunderstanding of the OSC NP formation mechanism by miniemulsion in the literature.

To better understand the conditions influencing direct extraction versus classic miniemulsion NP formation, we investigated a lower-energy emulsification approach using shear mixing. Since ultrasonication, a high-energy method, appears to favor direct extraction, we hypothesized that a lower-energy technique might promote the classic pathway and that adjusting the shear rate could regulate the balance between these two competing NP formation mechanisms. Performing shear-mixing emulsification with the model P3HT@SDS system yielded emulsions that separated over 24 h (see Figure S16), and using different rotational speeds revealed a clear dependence of the yield of direct extraction NPs on emulsification energy input. For rotational speeds less than 1000 rpm the formation of stable CHCl_3_ droplets was not observed. As the rpm was increased from 1000 to 10,000, after allowing the CHCl_3_ droplets to settle, it could be clearly observed that the amount of P3HT directly extracted to the aqueous phase increased (see photograph in Figure 3a and UV–vis absorption data from the aqueous phase in Figure S17a). The NPs formed in the aqueous phase had similar sizes and dispersity across all rotational speeds and were comparable to those produced by ultrasonication, as shown in Figure S17b. However, the amount of OSC extracted to the aqueous phase (i.e., only 65% when shear mixing at 10,000 rpm for 5 min) was still considerably less than when using standard ultrasonication conditions (1 min 40% amplitude). To give some insight into this difference, we estimated the power imparted to the emulsion using both techniques by measuring the initial temperature increase rate (see Methods). Even at very low amplitude (1% max power, 50% duty cycle, using a 700 W ultrasonicator with 1/8″ microtip probe), ultrasonication delivers considerably more energy than achieved by shear mixing (at 10,000 rpm with a 3/8″ high-shear mixing head). As can be seen in Figure S18a the power received by the emulsion is almost 2 orders of magnitude higher for ultrasonication at 1% ampitude (3000 mW) compared to shear mixing at 10,000 rpm (70 mW). During ultrasonication, it is reasonable to conclude that the energy is both sufficiently high and sustained to ensure that most droplets are exposed to high-energy zones near the probe, enabling nearly complete extraction of OSCs into the aqueous phase within the time scale tested (Figure S14).

**Figure 3:**
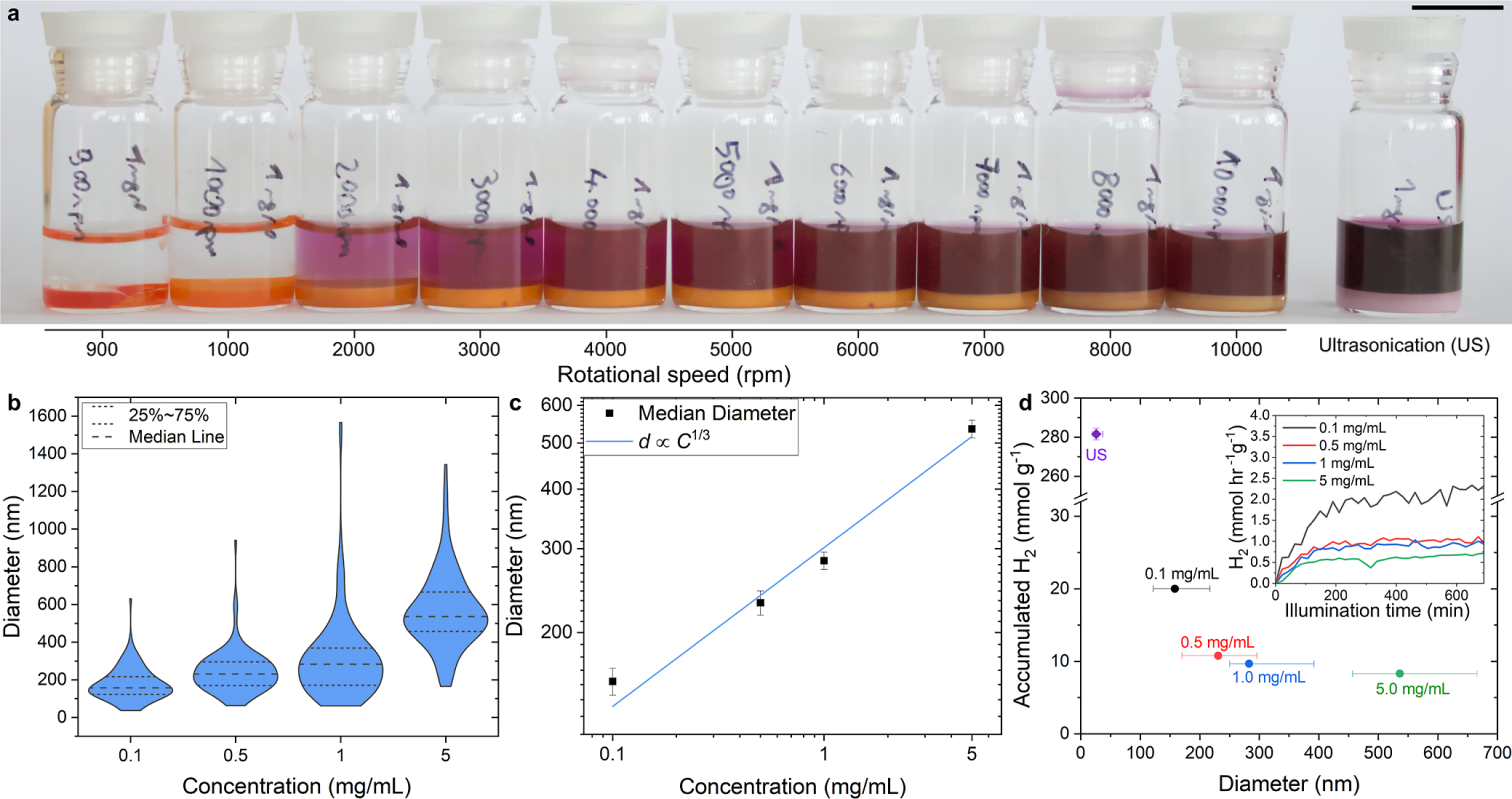
NP formation by shear mixing. (a) photographs of the CHCl_3_-in-water emulsions with P3HT@SDS (1 mg/mL P3HT, 5 mg/mL SDS) produced at different rotational speeds for 10 min, and for ultrasonication (40% amplitude, 1 min). CHCl_3_ droplets were allowed to settle. The scale bar is 15 mm. (b) Violin plot diameter distributions for the PTB7-Th:ITIC(3:7)@TEBS/SDS(3:1) NPs formed after separating and washing the bottom phase (emulsion) from the aqueous phase and evaporating the CHCl_3_, as measured by TEM. (c) Logarithmic plot of the median NP sizes vs OSC concentration for the samples in panel b. The blue line represents the best-fit curve with a slope of 1/3 (expected for the miniemulsion-evaporation pathway). The error bars indicate the median standard error in the NP diameters. (d) Total H_2_ production per gram of OSC integrated over 10 h of illumination. The symbols are placed at the median diameter and the horizontal error bars represent the first and third quartile of the size distribution. A standard ultrasonication sample made with the same mixture of surfactant (TEBS/SDS 3:1) (purple) has been added for comparison (see Figure S22). The inset plot shows the HER rates for the samples in panel b (nominal Pt loading = 16 wt % in weight, ascorbic acid concentration = 0.23 M).

In contrast, since shear mixing can also produce NPs by the direct extraction mechanism, it is reasonable to conclude that this process generates local energy spikes sufficient to trigger the extraction. However, the lower overall energy input results in a significantly decreased direct extraction rate. As a result, only a portion of the OSCs is extracted, with the remainder retained within the CHCl_3_ droplets. Indeed, over the conditions tested, the log of the weight fraction of OSC remaining in the CHCl_3_ phase scales inversely with the power input, as shown in Figure S18b. Similar results with shear mixing were obtained with the PTB7-Th:ITIC system, and emulsions could be formed while minimizing the rate of direct extraction, which allowed the formation of BHJ NPs via a true emulsion-evaporation mechanism (we note that a TEBS/SDS mixture at 3:1 was necessary to stabilize the emulsion during shear-mixing). Briefly, after washing the emulsion formed via shear mixing with an aqueous surfactant solution to remove any directly extracted NPs, BHJ NPs were formed from the OSC remaining in the CHCl_3_ droplets (see optical microscope image Figure S19), by CHCl_3_ evaporation. Direct analysis of the TEM images of the produced NPs at 3500 rpm (see representative images Figure S20) showed a clear dependence of the NP diameter on the starting OSC concentration in the organic phase as shown in the distributions presented in Figure 3b. We note that the median NP size was considerably larger, ranging from 150 to 530 nm when varying the OSC concentration from 0.1 to 5 mg/mL, compared to using ultrasonication and the direct extraction mechanism. Nevertheless, plotting the median diameter against the OSC concentration on a log–log scale confirms a fit to the cube-root model expected for a true emulsion-evaporation NP formation mechanism (Figure 3c). It should be also noted that the size of the directly extracted NPs present in the aqueous phase after shear mixing showed no dependence on the OSC concentration and were similar to those obtained by ultrasonication (See TEM images Figure S21), supporting the conclusion that they were formed following the same direct extraction pathway, which is not unique to ultrasonication.

Photocatalytic H_2_ evolution testing of the BHJ NPs produced via the true emulsion-evaporation approach showed that gas evolution rates decreased with increasing median NP size, in the size range tested (Figure 3d), likely due to the reduction in specific surface area and the increase in charge transport path length to the semiconductor/liquid interface. The best performing NPs, prepared from 0.1 mg/mL OSC solution (and with a median *d* of 150 nm) exhibited a peak H_2_ evolution rate of 2 mmol H_2_ hr^–1^ g^–1^ accumulating 20 mmol H_2_ per gram photocatalyst used over 10 h under operation. A maximum apparent quantum yield (AQY) of 1.2% at an illumination wavelength of 710 nm was also measured. In contrast NPs with an median *d* of 530 nm accumulated less than 10 mmol H_2_ per gram photocatalyst over 10 h. However, a BHJ NP sample prepared by direct extraction (ultrasonication) with the same surfactant mixture (TEBS/SDS 3:1) and a median size of 25 nm (see Figure S22) showed ∼14 times higher H_2_ production compared to the best “true emulsion- evaporation” case under the same testing conditions. This large photocatalytic activity difference could simply originate from the size dissimilarity. However, testing this by producing NPs with our emulsion-evaporation technique with a size comparable to the ultrasonication direct-extraction NPs proved to be unfeasible due to the requisite low OSC concentration and the resulting small OSC/surfactant ratio. While small NPs (<100 nm) are indeed formed using an OSC concentration of 5 μg/mL, the large ratio of OSC/surfactant (1:5000 by weight) complicated the evaluation of NP size by TEM, due to the presence of a thick surfactant film on the TEM grid (see Figure S23) or to detect H_2_ evolution. Indeed, despite dialyzing the dispersion for more than 180 h, no H_2_ was observed in photocatalytic testing. In addition, we cannot discount that changes in BHJ morphology, caused by the potentially very different kinetics of OSC aggregation between the two methods, do not contribute to the difference in performance. Thus, more work remains needed to further reduce the size and polydispersity of the NPs obtained with the true emulsion-evaporation approach while minimizing the fraction of directly extracted material, in order to reach optimal photocatalytic activity. Nevertheless, our approach demonstrates the possibility of tuning OSC NP size with an emulsion-evaporation method, enabling the first demonstration of the effect of BHJ NP size on photocatalytic H_2_ evolution activity.

Overall, tuning important parameters of OSC NPs, such as particle size, is critical for advancing their performance for numerous applications. For the application of photocatalysis, particle size has been expected to have a significant effect on performance. Indeed, small NPs should improve photocatalytic performance owing to a larger specific surface area, reduced light shadowing effects (optical absorption path length in typical OSC is ∼100 nm),^39,40^ and better charge extraction (carrier diffusion length in typical OSC is ∼100 nm).^41^ However, excessively small NPs can suffer from extensive recombination due to insufficient distance between the separated charge carriers and poor crystallinity of component materials. The true emulsion-evaporation NP formation mechanism introduced in this work has provided some important insight into the optimal size for BHJ NP photocatalysts for solar-driven H_2_ evolution, confirming that diameters less than 100 nm are needed for the PTB7-Th:ITIC system.

Moreover, to the best of our knowledge, our work is the first to identify the formation of OSC NPs directly during emulsification and prior to organic solvent evaporation. While a detailed mechanism for this direct extraction phenomenon is yet to be elucidated, we hypothesize that interfacial perturbations during emulsification may play an important role. The direct extraction of hydrophobic or hydrophilic components from a good solvent dispersed in a continuous phase of an antisolvent has been previously reported. In such systems, interfacial instabilities achieved under conditions of low interfacial tension^42–44^ or the formation of small micelles^45,46^ facilitate the transfer of components from good solvent droplets to the continuous antisolvent phase. In the systems studied here, interfacial tension is significantly higher. However, the high energy input used during emulsification to produce miniemulsion, may induce interfacial instabilities, leading to a similar extraction mechanism. The direct extraction of OSC from the interface could explain the observed independence of NP size on OSC concentration. According to this hypothesis, only OSC molecules at or near the interface would contribute to NP formation, and NP size would not correlate with OSC concentration in the bulk of the droplet. It is plausible that, even at the lowest concentrations tested, the organic/aqueous interface was already saturated with OSC molecules, resulting in a consistent interfacial population and, consequently, similar NP sizes.

## Conclusions

This work reported, for the first time, the occurrence of the phenomenon of direct extraction of organic semiconductors (OSC) from an organic to an aqueous phase during high-energy emulsification, prior to organic solvent evaporation, highlighting a significant gap in the understanding of OSC nanoparticle (NP) formation. Ultrasonication experiments with both P3HT and PTB7-Th:ITIC systems provided strong evidence for the existence of direct extraction as a competitive OSC NP formation pathway which dominates over the classical miniemulsion-evaporation mechanism at high emulsification energies, explaining the lack of particle size control in the bulk-heterojunction (BHJ) NP literature. Using lower-energy shear mixing emulsification instead of ultrasonication, the direct extraction pathway was partially suppressed to enable NP formation through a true emulsion-evaporation mechanism, yielding tunable NP sizes and enabling the first demonstration of the effect of BHJ NP size on photocatalytic H_2_ evolution activity. This work represents an important step toward the improvement of the photocatalytic H_2_ evolution activity of BHJ NPs through the optimization of particle size, complementary to previous research efforts to optimize blend composition and morphology. Future work will focus on elucidating the molecular mechanism of the direct extraction phenomenon, and on achieving smaller NP sizes and narrower size distributions, which are expected to yield higher photocatalytic activities.

## Methods

### Materials

PTB7-Th (poly­[4,8-bis­(5-(2-ethylhexyl)­thiophen-2-yl)­benzo­[1,2-b;4,5-b’]­dithiophene-2,6-diyl-*alt*-(4-(2-ethylhexyl)-3-fluorothieno­[3,4-*b*]­thiophene-)-2-carboxylate-2–6-diyl], also known as PBDTTT-EFT or PCE10), molecular weight 125,205 g/mol, ITIC ((3,9-Bis­(2-methylene-(3-(1,1-dicyanomethylene)-indanone))-5,5,11,11-tetrakis­(4-hexylphenyl)-dithieno­[2,3-d:2′,3′-d′]-*s*-indaceno­[1,2-b:5,6-b’]-dithiophene))), and P3HT (poly­(3-hexylthiophene)), molecular weight 60,150 g/mol, were purchased from Ossila Ltd., and used as received. SDS (Sodium dodecyl sulfate) was purchased from Chemie Brunschwig AG. Ascorbic acid, ACS reagent grade, was purchased from Sigma-Aldrich and used without further processing. Platinum precursor K_2_PtCl_6_, was purchased from Sigma-Aldrich (≥99.99%). Chloroform (CHCl_3_) and chlorobenzene (CB) were purchased from EMD Millipore Corporation. Surfactant solutions were prepared in ultrapure water (from a VWR P Series Ultrapure Water System). Dispersions of polystyrene nanoparticles with nominal diameter 20 nm, 50 nm, 100 nm, 300 nm were purchased from Abvigen Inc.

### TEBS Synthesis

(2-(3-thienyl)­ethyloxybutylsulfonate sodium salt) was synthesized according to the following procedure. Sodium hydride (60% in paraffine oil, 1.11 g, 27.8 mmol, 1 eq., TCI) was introduced in an oven-dried two-neck flask of 250 mL. A condenser was placed on top and a rubber septum on its side before careful inertization of the reactor with argon. Through the septum was injected dry tetrahydrofuran (99.5%, 90 mL, Thermo Scientific) followed by the dropwise addition of 2-(3-thienyl)­ethanol (98%, 3.5 mL, 30.5 mmol, 1.1 eq., ABCR). The mixture was stirred for 30 min and then cooled down with an ice bath. At this stage, 1,4-butane sultone (99%, 4.02 mL, 38.9 mmol, 1.4 eq., Aldrich) was added drop by drop through the septum. The mixture was refluxed for 16 h yielding a cloudy solution. The precipitate was isolated by centrifugation and redispersed in 40 mL of acetone. The tube was sonicated for 5 min to ensure the good washing of the solid and then centrifugated again. This process was repeated two more times, once with acetone and once with diethyl ether. The resulting solid was dried under vacuum to yield 7.5 g of TEBS as a white-beige solid with a yield of 84%. ^1^H NMR (400 MHz, D_2_O): δ 7.4–7.28 (1H, dd), 7.17–7.06 (1H, s), 7.06–6.70 (1H, d), 3.74–3.65 (2H, t), 3.51–3.44 (2H, t), 2.89–2.77 (4H, m), 1.71–1.56 (4H, m). ^13^C NMR (101 MHz, D_2_O): δ 139.37, 128.42, 126.07, 121.62, 70.36, 69.88, 50.65, 29.58, 27.40, 20.89.

### NP Formation by Ultrasonication

Organic semiconductor precursor solutions were prepared at different concentrations in CHCl_3_. The solutions were heated at 40 °C several hours to help dissolving the organic semiconductors. For the formation of bulk heterojunction NPs, PTB7-Th and ITIC precursor solutions were mixed in a 3:7 weight ratio and stirred at 40 °C for at least 3 h before being used. 400 μL of precursor solution were placed in a 6 mL vial and 2 mL of surfactant (TEBS or SDS)-water solution were added to the vial. Ultrasonication was applied by a Qsonica Q700 Sonicator with 1/8″ microtip probe (tip diameter 3.2 mm). The microtip probe was inserted in the vial, slightly above the organic semiconductor solution at the bottom, and the vial was placed in an ice/water bath to prevent excessive heating during sonication. If not specified, the solution was sonicated for 1 min at 40% of the maximum amplitude with 10 s pulsed sonication periods and 10 s intervals between each pulse. The maximum (100%) amplitude for the setup used in this work is 380 μm. In the cases where CHCl_3_ was evaporated, after ultrasonication, the samples were heated at 70 °C under nitrogen gas flow with magnetic stirring, for at least 30 min. Dialysis was carried out in dialysis cassettes with a 3500 kDa molecular weight cutoff membrane (Slide-A-Lyzer from Thermo Scientific). The solution was injected in the cassette and the cassette was immersed in 1 L of ultrapure water under magnetic stirring. The water was changed at least 3 times at 3, 12, and 24 h intervals.

### NP Formation by Shear Mixing

A high shear mixer (Silverson L5M-A) was used with a 3/8″ shear mixing head. The mixing head was introduced in the solution in a 5 mL vial. Different times and rotation speed were used in this work and the parameters are mentioned with the corresponding results. The volume of solution and the organic/aqueous solvent ratio were kept the same as in ultrasonication. If not specified, the mixing time was 5 min at 3500 rpm, the OSC concentration was 1 mg/mL and the surfactant concentration was 5 mg/mL. For the formation of NPs only from the CHCl_3_ droplets from shear mixing, after letting the CHCl_3_ settle at the bottom of the vial, the aqueous phase (2 mL TEBS/SDS, 3:1 ratio, 5 mg/mL) was extracted and 3 mL of fresh surfactant solution were added. The vial was gently swirled to mix the droplets with the fresh surfactant solution, and the CHCl_3_ droplets were left to settle at the bottom. The process was repeated several times until the aqueous phase became colorless. Then, CHCl_3_ was evaporated by heating the solution at 70 °C under nitrogen flow and magnetic stirring, for at least 30 min. Dialysis was carried out as described for ultrasonication NPs. For the NPs formed with an OSC concentration of 5 μg/mL, the dialysis was conducted for 8 days changing the water after 3 h and then every 12 h.

### Dynamic Light Scattering Measurements

Size measurements were carried out in plastic cuvettes (ZEN0040) using a Zetasizer Pro blue label instrument (Malvern Panalytical), with a laser wavelength of 632.8 nm, and the scattered signal was measured at an angle of 173°. The size of commercial polystyrene nanoparticles was measured using both TEM and DLS. The comparison between the two techniques allowed us to assess the differences in size measurement for organic nanoparticles with narrow size distribution (See Figure S11). TEM provides direct imaging of the dry particles, while DLS offered hydrodynamic size estimates in suspension. This evaluation shows the differences between the two methods on the observed particle size. The violin plots used to display the size distribution of the samples were normalized by width and plotted with the Silverman’s Kernel bandwidth.

### Transmission Electron Microscopy Imaging

TEM samples were prepared by drop-casting diluted aqueous NP dispersions on TEM grids (∼3 nm amorphous carbon on 400 μm copper mesh, Ted Pella Inc., USA) followed by drying for at least 12 h under vacuum. TEM and STEM images were taken on a Thermo Fisher Scientific Talos F200S microscope operated at 200 kV using a Ceta camera. The size of the nanoparticles was measured manually using the ImageJ software from FIJI. For the nanoparticles formed with miniemulsion over 1000 particles were measured, and around 100 nanoparticles in the case of the nanoparticles form with shear mixing using the chloroform macroemulsion. The violin plots used to display the size distribution of the samples were normalized by width and plotted with the Silverman’s Kernel bandwidth.

### Optical Microscopy

Optical microscopy images were acquired using a Nikon Eclipse LV100ND microscope equipped with a camera (model DFK 72AUC02 from The Imaging Source). After formation of the emulsion by ultrasonication or shear mixing the emulsion was drop cast on a microscope glass slide and a cover glass was deposited on top of the solution.

### Ultraviolet–Visible (UV–vis) Spectroscopy

UV–vis spectroscopy data was obtained using a PerkinElmer Lambda 365 UV–vis spectrophotometer with a 1 nm slit width and scan rate of 1 or 2 nm/s, in 10 mm path length quartz cuvettes. To measure the UV–vis spectra of the aqueous phase after emulsification, CHCl_3_ was let to settle at the bottom of the vial for 24 h. Then the aqueous phase was diluted to have a maximal absorbance below 1. The OSC concentration in CHCl_3_ phase was quantified by UV–vis spectroscopy after centrifugation and interpolation in a calibration curve of standard CHCl_3_ solutions of known OSC concentration, using the absorbance at 680 nm and following Beer-Lamber law (Figure S14). The absorption spectrum of the PTB7-Th:ITIC@TEBS/SDS NPs made by shear mixing was measured using a UV–vis spectrometer (UV-3600 SHIMADZU) equipped with an integrating sphere, in a 1 mm path length quartz cuvette.

### Photocatalytic Performance Measurements

Hydrogen evolution rates were measured using a purge reactor with direct injection to a gas chromatograph (GC). Three mL of NP dispersion (0.2 mg of OSC) were charged into a 4 mL glass vial with a septum and further sealed with PTFE and parafilm to prevent any leaks. Ascorbic acid was added as the sacrificial electron donor at a concentration of 0.23 M. In situ Pt photodeposition was achieved by adding a small aliquot of aqueous K_2_PtCl_6_ solution (10 mg/mL) to have a nominal Pt loading of 16% in weight relative to the OSC. The reactor was purged with nitrogen at 6 mL/min before and during illumination. The reactor was purged for ∼1 h in the dark until a stable oxygen peak was observed by GC. Subsequently, the dispersion was illuminated with a 500 W Xe lamp fitted with a KG2 Colored Glass Bandpass Filter (304–785 nm, ThorLabs) at 1 Sun irradiance. A Nexis GC-2030 gas chromatograph (Shimadzu) fitted with a BID detector and customized with an automatized injection system (Chemlys SAS, France) was used to quantify the amount of hydrogen evolved, with injections at 20 min intervals. For the PTB7-Th:ITIC@TEBS/SDS NPs prepared by shear mixing and ultrasonication, the samples were diluted or concentrated in order to have the same absorption at 740 nm.

### Apparent Quantum Yield

AQY measurements were performed using the system described above with the addition of a monochromator (Quantum design MSH300F, fwhm 20 nm). Before the measurement, the sample was subjected to illumination under 1 sun illumination, similarly to hydrogen evolution measurements for 2 h for Pt photodeposition. The average HER rate over 10 h of illumination was converted into an electron flux and compared to the incident photon flux to estimate the AQY using eq 1, where *nH*_2_ denotes the number of moles of electrons circulating per hour, and *n* photons represents the total number of photons incident on the reactor per hour. The wavelength was set to 710 nm and the irradiance to 1.2 mW/cm^2^.

1 $$AQY\left(\%\right)=\frac{2\times nH_{2}}{nphotons}\times 100$$

### Relation between Concentration and NP Diameter for a Classic Miniemulsion-Evaporation

The number of OSC molecules in one emulsion droplet, *N*_molecule_, can be calculated using the volume of the droplet, *V*_droplet,_ and the OSC concentration of the organic solution, *C*, as

2 $$N_{molecule}=V_{droplet}\times C$$

The volume of the NP formed after evaporation of the organic solvent in said droplet, *V*_NP_, is given by the density of the OSC molecules in the NP, ρ, as

3 $$V_{NP}=\frac{N_{molecule}}{\rho }$$

Substituting *N*_molecule_ from eqs 2 into [Disp-formula eq3], and using the relation between the volume of a sphere, *V*, and its diameter, *d*

4 $$V=\frac{4}{3}\pi {\left(d/2\right)}^{3}$$

we find the relation between the diameter of the NP, *d*, and the diameter of its parent droplet, *D*

5 $$d=D{\left(C/\rho \right)}^{1/3}$$

### Measure of Energy Input during Shear Mixing and Ultrasonication

The power input during emulsification was estimated by measuring the initial temperature increase rate (d*T*/d*t*) during ultrasonication or shear mixing. A thermocouple was inserted into a vial identical to those used for NPs formation, containing chloroform and a SDS aqueous solution (5 mg/mL) in the same volume ratio as used for NPs formation. The vial was placed in a thermally insulating PVC block, and temperature increase as a function of time was recorded. Two control vials containing water were used: one placed in the same insulating block without energy input to verify minimal heat loss, and one left nearby to monitor and correct for ambient temperature drift. The input power, *P*, was then estimated using the following relation

6 $$P=mc_{avg}\frac{dT}{dt}$$

where *m* is the total mass of the liquid and *c*_avg_ is a mass fraction-averaged specific heat capacity. For shear mixing, the power input was estimated from the temperature increase rate over the first 5 min of mixing. For ultrasonication, the power was calculated from the initial 10 s of sonication. This value was then adjusted in consideration of the sonication duty cycle (10 s on/10 s off).

## Supplementary Material



## Notes

Tables S1 and Figures S1–S23 as described in the main text. The main data supporting the findings of this study within the manuscript and its associated Supporting Information are available via repository at doi: 10.5281/zenodo.15261652 (PDF)
