# Supplementary material for: Unexpected Pathway in Organic Semiconductor Nanoparticle Formation
Source: ACS Nano. 2025 Jul 31;19(31):28469–77. doi: 10.1021/acsnano.5c07335 (PMC12356114; doi:10.1021/acsnano.5c07335)
Supplement: Supplementary file 1 [file nn5c07335_si_001.pdf]

Supporting information for

Unexpected pathway in organic semiconductor nanoparticle formation

*Arthur E. Bouchez[‡], Connor R. Firth[‡], Arnau Bertran, Colin Jeanguenat, Jun-Ho Yum, Kevin Sivula**

Laboratory for Molecular Engineering of Optoelectronic Nanomaterials, Institute of Chemical Sciences and Engineering, École Polytechnique Fédérale de Lausanne (EPFL), Lausanne, CH-1015, Switzerland.

*kevin.sivula@epfl.ch

‡ A.E.B. and C.R.F. authors contributed equally.

1 Supplementary tables

Table S1. *Short overview of selected published works for OSC NP formed by the miniemulsion-evaporation method with ultrasonication.*

OSC (conc)	Surfactant (conc)	Diameter	Characterization	Ref
P3HT (30mg/mL)	SDS (2.3mg/mL)	~140nm	DLS/SEM	[1]
P3HT/$PC_{61}BM$ (53mg/mL)	SDS (12mg/mL)	~80nm	DLS	[2]
P3HT/$PC_{61}BM$ (15mg/mL)	TEBS (5.6mg/mL)	~85nm	DLS/SEM	[3]
PTB7-Th :EH-IDTBR (0.5mg/mL)	TEBS (5mg/mL)	~80nm	DLS	[4]
PTB7-Th :EH-IDTBR (0.5mg/mL)	SDS (5mg/mL)	~45-65nm	DLS	[4]
PBDB-T :ITIC (0.5mg/mL)	SDBS (0.3-1.3mg/mL)	~40-100nm	DLS	[5]
PM6 :Y6 (0.5mg/mL)	TEBS (5mg/mL)	~100nm	DLS	[6]
Y6 (3mg/mL)	SDS (13.75mg/mL)	~50nm	DLS	[7]
Y6 (3mg/mL)	TEBS (13.75mg/mL)	~80nm	DLS	[7]
PTQ10 :Y6 (25mg/mL)	SDS (5mg/mL)	~90nm	DLS	[8]
P3HT (10mg/mL)	SDS(0.3-3mg/mL)	~45-120nm	DLS	[9]
$PC_{71}BM$:PCDTBT (5-50 mg/mL)	SDS(10-30mg/mL)	~30-330nm	TEM	[10]
PDPP5T/$PC_{61}BM$ (40mg/mL)	SDS(3-12mg/mL)	~30-60nm	DLS	[11]
P3HT/$PC_{61}BM$ (10-45mg/mL)	SDS(1.5-80mg/ml)	~30-150nm	DLS	[12]

(1) Tan, B.; Li, Y.; Palacios, M. F.; Therrien, J.; Sobkowicz, M. J. Effect of Surfactant Conjugation on Structure and Properties of Poly(3-Hexylthiophene) Colloids and Field Effect Transistors. *Colloids and Surfaces A: Physicochemical and Engineering Aspects* **2016**, *488*, 7–14. https://doi.org/10.1016/j.colsurfa.2015.10.002.

(2) Schwarz, K. N.; Farley, S. B.; Smith, T. A.; Ghiggino, K. P. Charge Generation and Morphology in P3HT : PCBM Nanoparticles Prepared by Mini-Emulsion and Reprecipitation Methods. *Nanoscale* **2015**, *7* (47), 19899–19904. https://doi.org/10.1039/C5NR06244F.

(3) Chowdhury, R.; Holmes, N. P.; Cooling, N.; Belcher, W. J.; Dastoor, P. C.; Zhou, X. Surfactant Engineering and Its Role in Determining the Performance of Nanoparticulate Organic Photovoltaic Devices. *ACS Omega* **2022**, *7* (11), 9212–9220. https://doi.org/10.1021/ACSOMEGA.1C05711/ASSET/IMAGES/LARGE/AO1C05711_0007.JPEG.

(4) Kosco, J.; Bidwell, M.; Cha, H.; Martin, T.; Howells, C. T.; Sachs, M.; Anjum, D. H.; Gonzalez Lopez, S.; Zou, L.; Wadsworth, A.; Zhang, W.; Zhang, L.; Tellam, J.; Sougrat,

R.; Laquai, F.; DeLongchamp, D. M.; Durrant, J. R.; McCulloch, I. Enhanced Photocatalytic Hydrogen Evolution from Organic Semiconductor Heterojunction Nanoparticles. *Nat. Mater.* **2020**, *19* (5), 559–565. https://doi.org/10.1038/s41563-019-0591-1.

(5) Yang, Y.; Li, D.; Wang, P.; Zhang, X.; Zhang, H.; Du, B.; Guo, C.; Wang, T.; Liu, D. Polymer/Non-Fullerene Acceptor Bulk Heterojunction Nanoparticles for Efficient Photocatalytic Hydrogen Production from Water. *Polymer* **2022**, *244*, 124667. https://doi.org/10.1016/j.polymer.2022.124667.

(6) Kosco, J.; Gonzalez-Carrero, S.; Howells, C. T.; Fei, T.; Dong, Y.; Sougrat, R.; Harrison, G. T.; Firdaus, Y.; Sheelamanthula, R.; Purushothaman, B.; Moruzzi, F.; Xu, W.; Zhao, L.; Basu, A.; De Wolf, S.; Anthopoulos, T. D.; Durrant, J. R.; McCulloch, I. Generation of Long-Lived Charges in Organic Semiconductor Heterojunction Nanoparticles for Efficient Photocatalytic Hydrogen Evolution. *Nat Energy* **2022**, *7* (4), 340–351. https://doi.org/10.1038/s41560-022-00990-2.

(7) Dolan, A.; De La Perrelle, J. M.; Milsom, E. R.; Small, T. D.; Metha, G. F.; Pan, X.; Andersson, M. R.; Huang, D. M.; Kee, T. W. *Surfactant Effects on Hydrogen Evolution by Small Molecule Non-Fullerene Acceptor Nanoparticles*.

(8) Holmes, A.; Laval, H.; Guizzardi, M.; Maruzzo, V.; Folpini, G.; Barbero, N.; Deniau, E.; Schmutz, M.; Blanc, S.; Petrozza, A.; Paternò, G. M.; Wantz, G.; Chambon, S.; Lartigau-Dagron, C.; Bousquet, A. Water-Based Solar Cells over 10% Efficiency: Designing Soft Nanoparticles for Improved Processability. *Energy Environ. Sci.* **2024**, *17* (3), 1107–1116. https://doi.org/10.1039/D3EE03744D.

(9) Satapathi, S.; Gill, H. S.; Li, L.; Samuelson, L.; Kumar, J.; Mosurkal, R. Synthesis of Nanoparticles of P3HT and PCBM for Optimizing Morphology in Polymeric Solar Cells. *Applied Surface Science* **2014**, *323*, 13–18. https://doi.org/10.1016/j.apsusc.2014.07.175.

(10) Parrenin, L.; Laurans, G.; Pavlopoulou, E.; Fleury, G.; Pecastaings, G.; Brochon, C.; Vignau, L.; Hadziioannou, G.; Cloutet, E. Photoactive Donor–Acceptor Composite Nanoparticles Dispersed in Water. *Langmuir* **2017**, *33* (6), 1507–1515. https://doi.org/10.1021/acs.langmuir.6b04496.

(11) Colberts, F. J. M.; Wienk, M. M.; Janssen, R. A. J. Aqueous Nanoparticle Polymer Solar Cells: Effects of Surfactant Concentration and Processing on Device Performance. *ACS Appl. Mater. Interfaces* **2017**, *9* (15), 13380–13389. https://doi.org/10.1021/acsami.7b00557.

(12) Ghazy, O.; Freisinger, B.; Lieberwith, I.; Landfester, K. Tuning the Size and Morphology of P3HT/PCBM Composite Nanoparticles: Towards Optimized Water-Processable Organic Solar Cells. *Nanoscale* **2020**, *12* (44), 22798–22807. https://doi.org/10.1039/D0NR05847E.

2 Supplementary figures

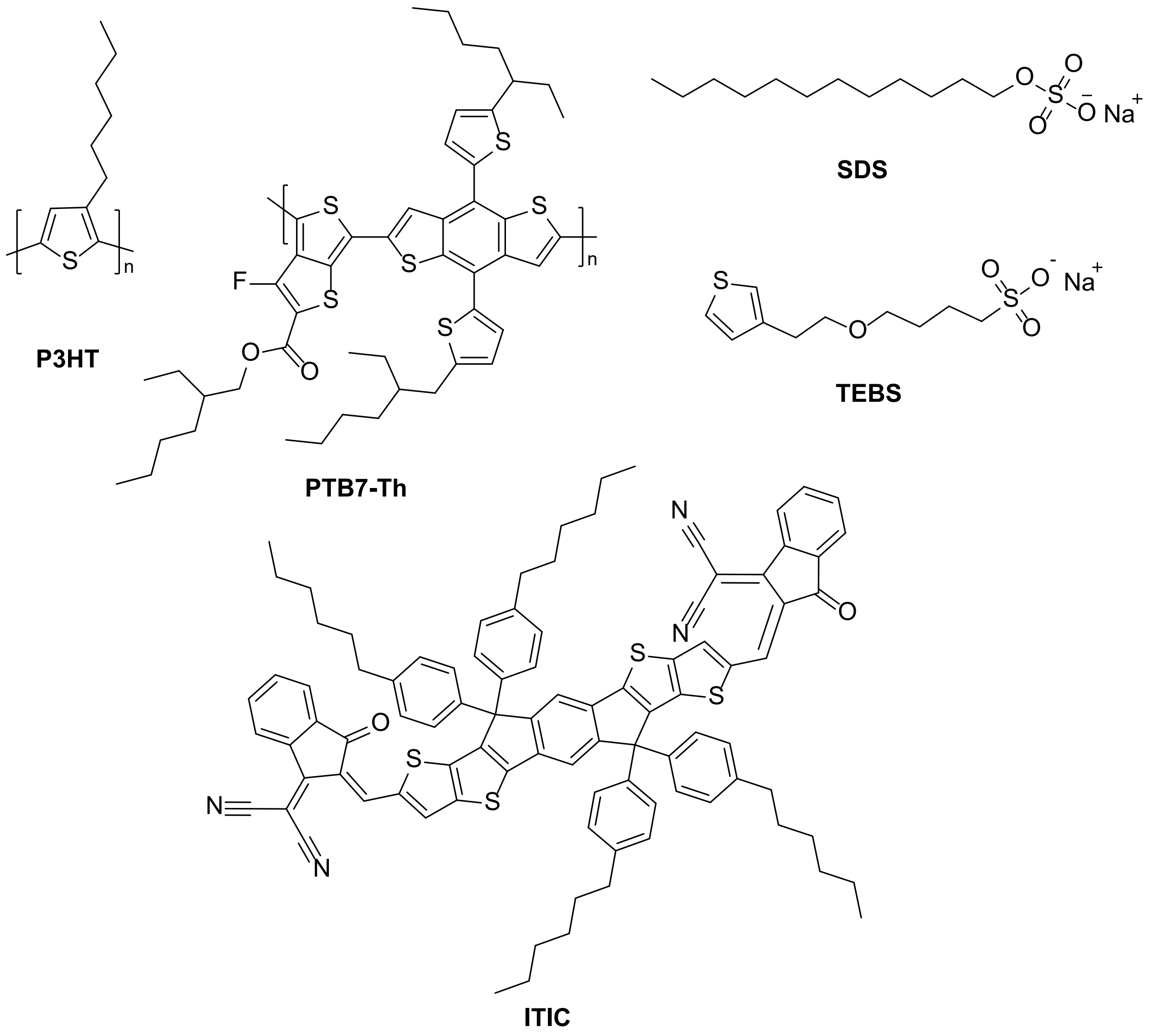

Figure S1. Chemical structures of the molecules used in this work.

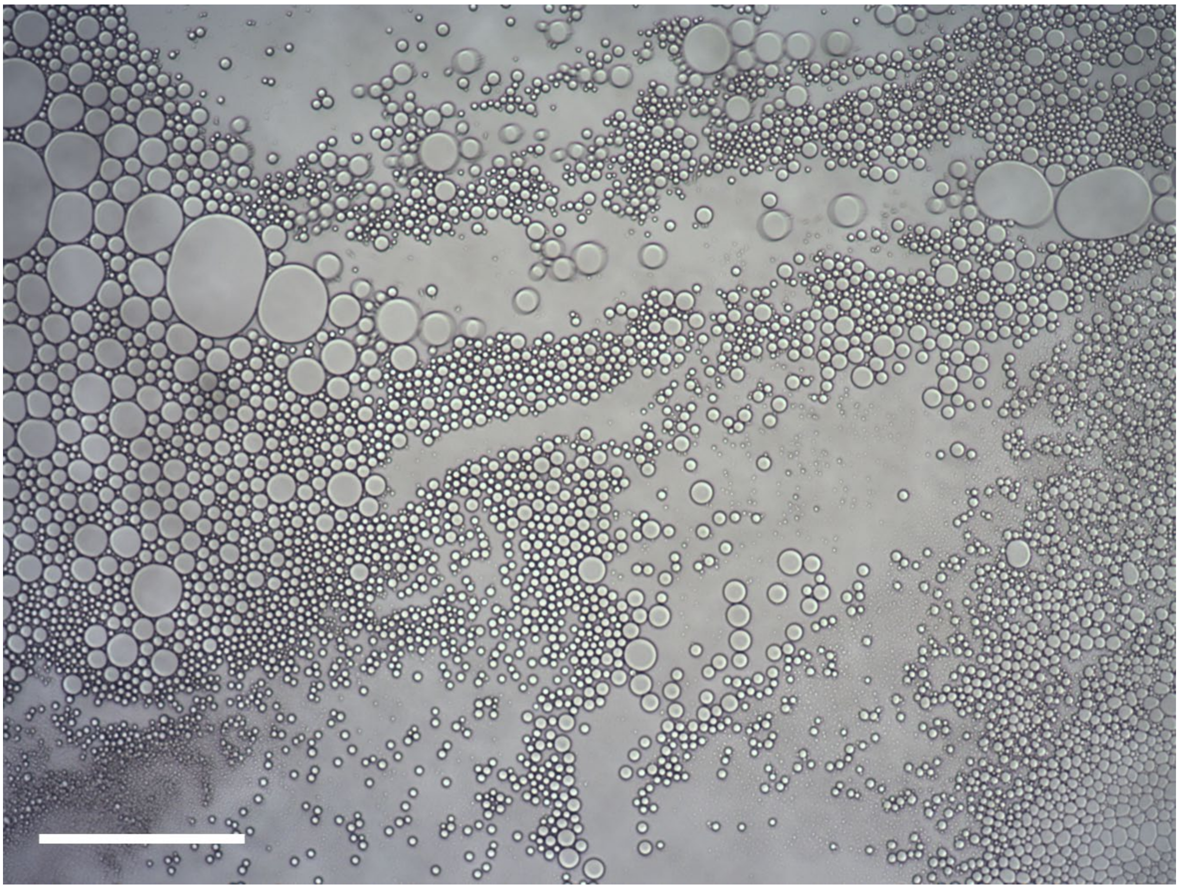

Figure S2. Optical microscope image of the emulsion droplets obtained after ultrasonication for a P3HT/$CHCl_3$ (droplets) in water/SDS (continuous phase) emulsion at a P3HT concentration of 5 mg/mL and a surfactant concentration of 5 mg/mL. The scale bar is 50 µm.

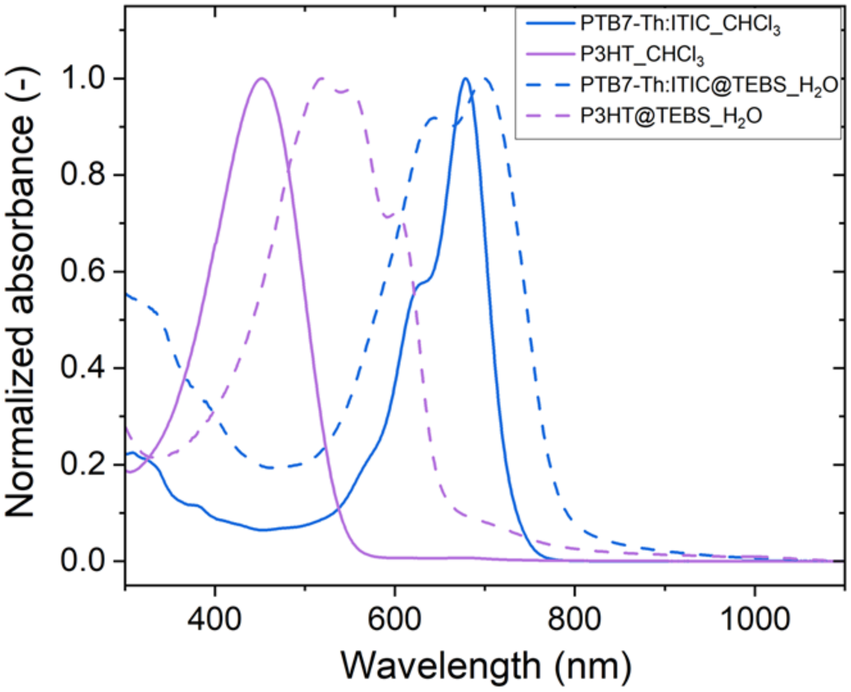

Figure S3. UV-Vis spectra of P3HT (purple) and PTB7-Th:ITIC (blue) in $CHCl_3$ and as NPs solution in water (dashed). The NPs were collected in the aqueous phase during

ultrasonication after letting the $CHCl_3$ settle at the bottom of the vial. The NPs were formed with 5mg/mL OSCs and 5mg/mL of TEBS.

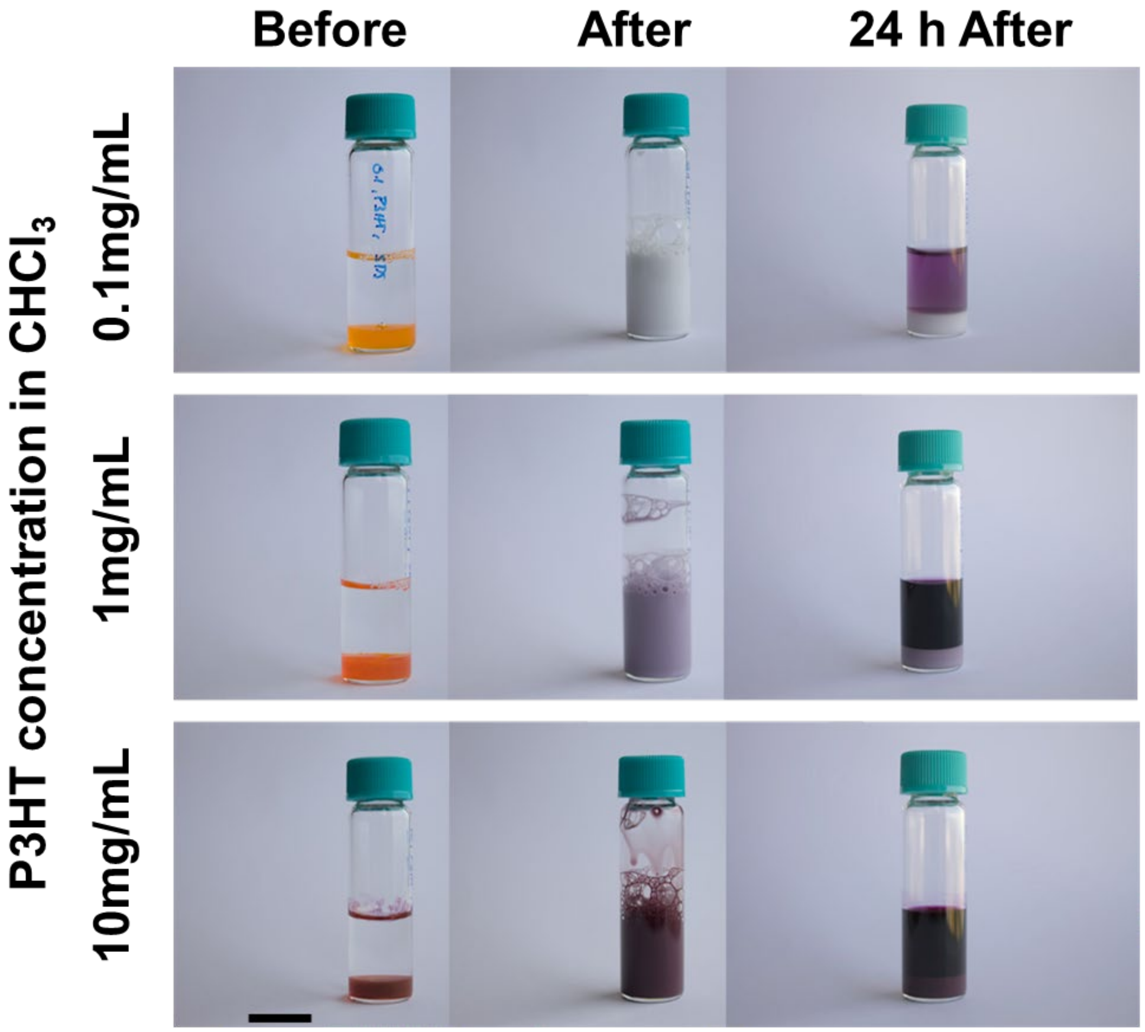

Figure S4. Photographs of the P3HT@SDS emulsion system (SDS concentration was 5 mg/mL in water) for varying concentrations of P3HT in $CHCl_3$ at different points in the preparation process (before, immediately after ultrasonication, and 24 h after ultrasonication), no heating was applied to remove the $CHCl_3$. The scale bar is 16 mm.

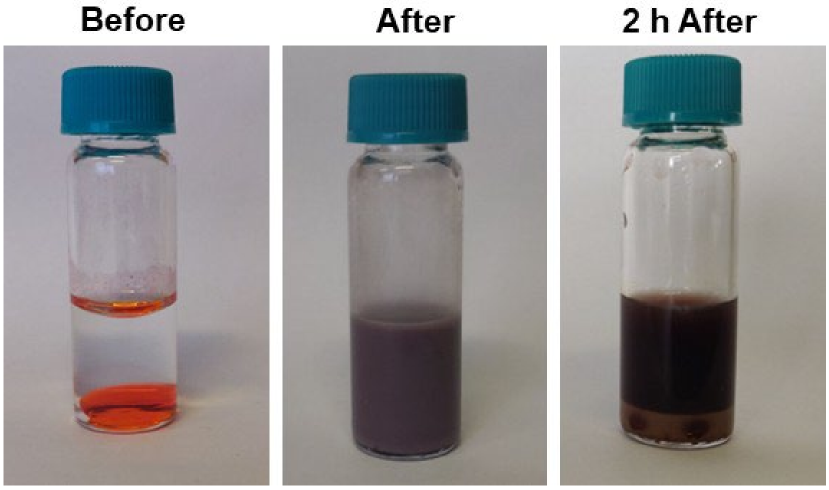

Figure S5. Photographs of the P3HT@SDS emulsion system (without added SDS) with P3HT in $CHCl_3$ (P3HT concentration 5 mg/mL) at different points in the preparation process (before, immediately after ultrasonication, and 24 h after ultrasonication), no heating was applied to remove the $CHCl_3$.

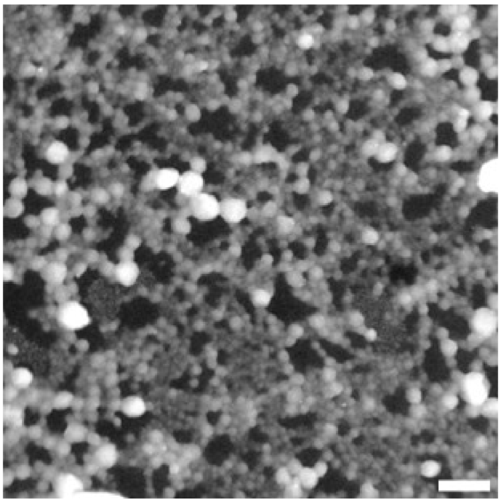

Figure S6. STEM image of the P3HT NPs formed in the aqueous phase during ultrasonication without surfactant (P3HT concentration 5 mg/mL), after letting the $CHCl_3$ settle at the bottom of the vial. The scale bar is 100nm.

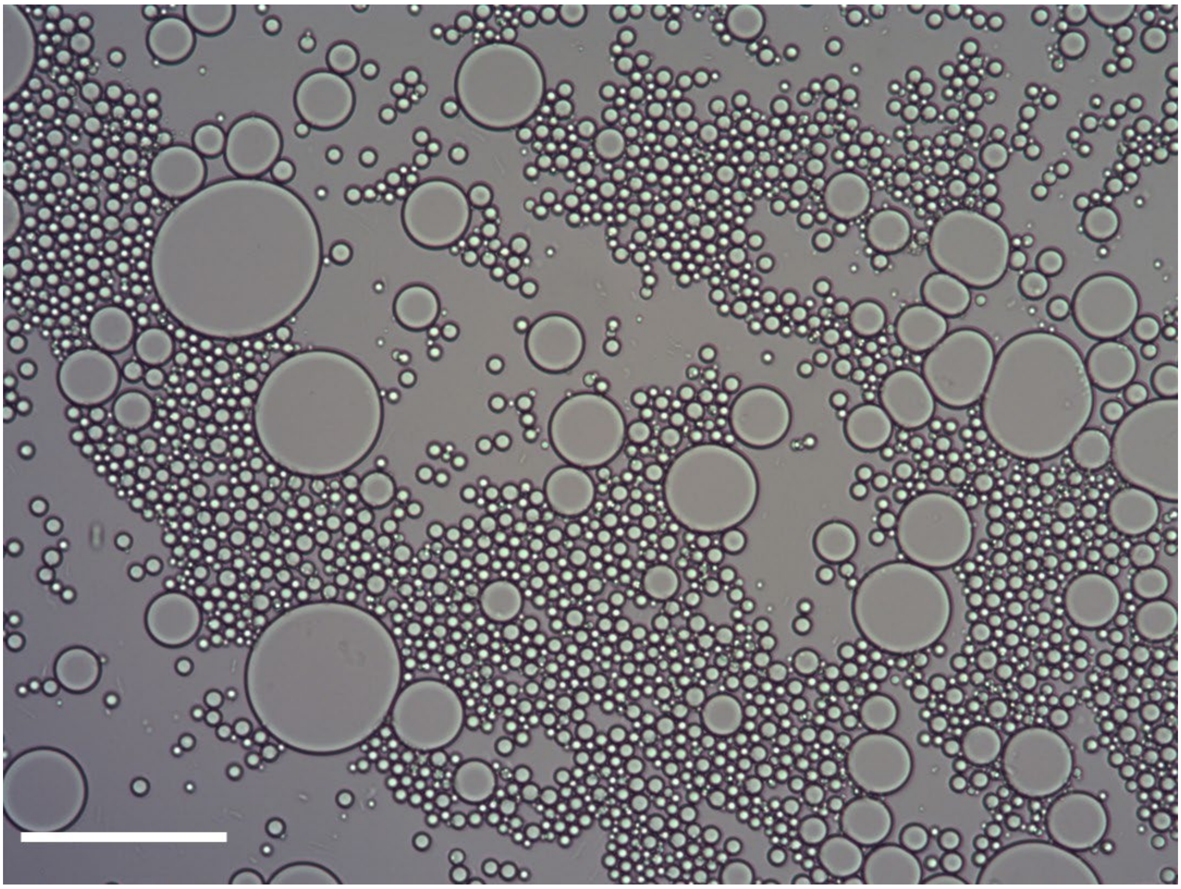

Figure S7. Optical microscope image of the emulsion droplets obtained after ultrasonication for a PTB7-Th:ITIC/$CHCl_3$ (droplets) in water/TEBS (continuous phase) emulsion at an OSC concentration of 5 mg/mL and a surfactant concentration of 5 mg/mL. The scale bar is 50 µm.

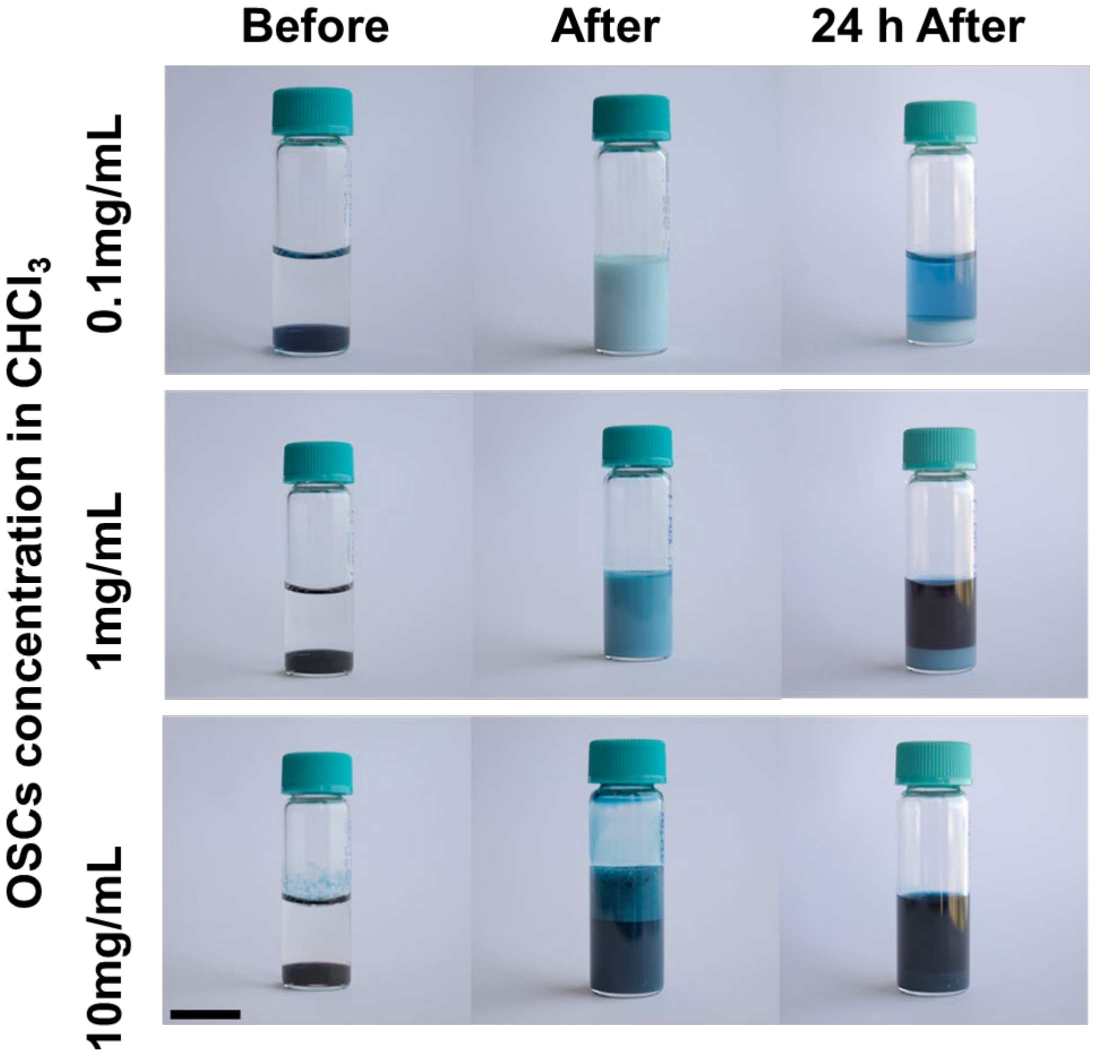

Figure S8. Photographs of the PTB7-Th:ITIC(3:7)@TEBS emulsion system (TEBS concentration was 5 mg/mL in water) for varying concentrations of OSCs in $CHCl_3$ at different points in the preparation process (before, immediately after ultrasonication, and 24 h after ultrasonication), no heating was applied to remove the $CHCl_3$. The scale bar is 16 mm.

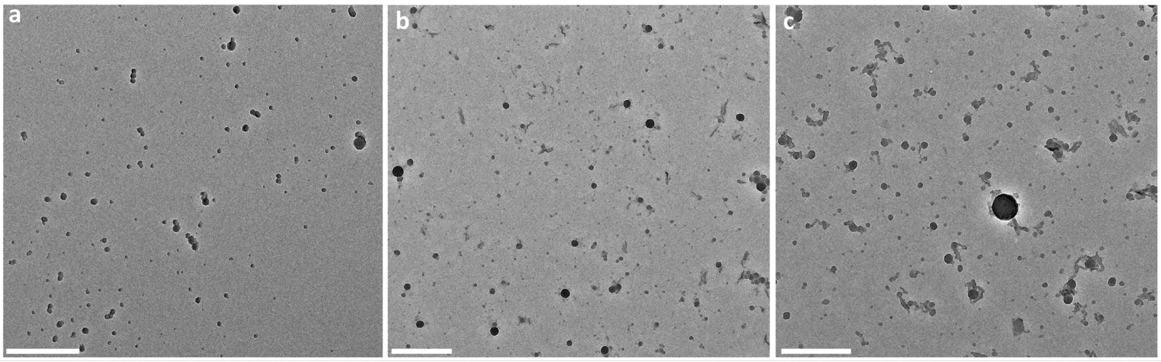

Figure S9. Examples of TEM images of PTB7-Th:ITIC@TEBS NPs prepared with different OSC concentrations: **a)** 0.1 mg/mL, **b)** 1 mg/mL, and **c)** 10 mg/mL. The TEBS concentration was 5 mg/mL and the NPs were collected from the aqueous phase of the mixture without evaporating $CHCl_3$, after letting it settle at the bottom of the vial. The scale bars are 500 nm.

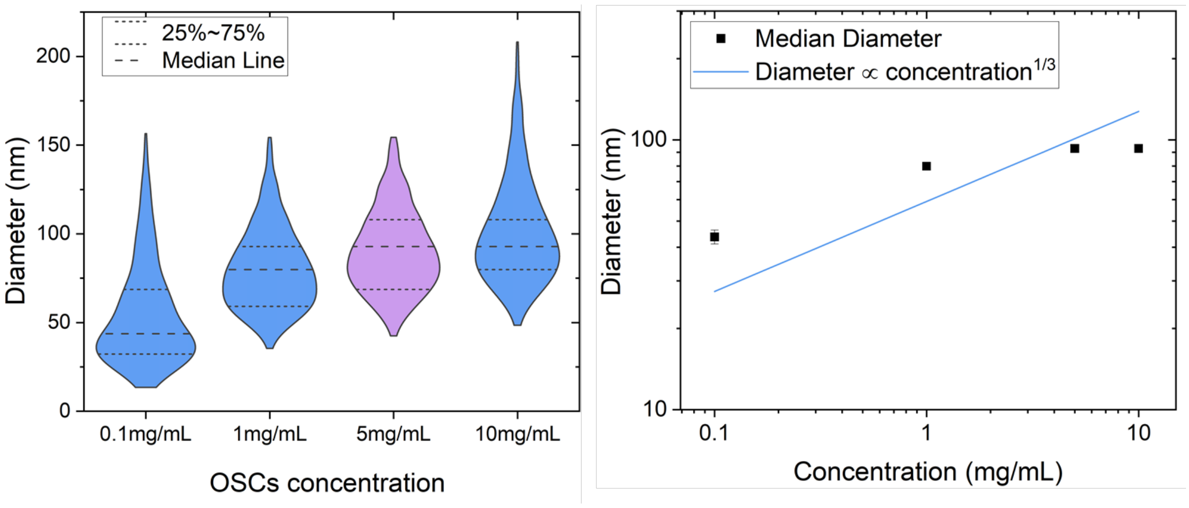

Figure S10. Size distributions for the PTB7-Th:ITIC@TEBS NPs from the aqueous phase (without evaporation of $CHCl_3$) measured by DLS. The NPs were formed with: **a)** Different OSC concentrations, and **b)** shown on a log-log plot in comparison to the expected cube root trend. The purple distribution represents the "standard" sample (see Figures S11 and S12 below)

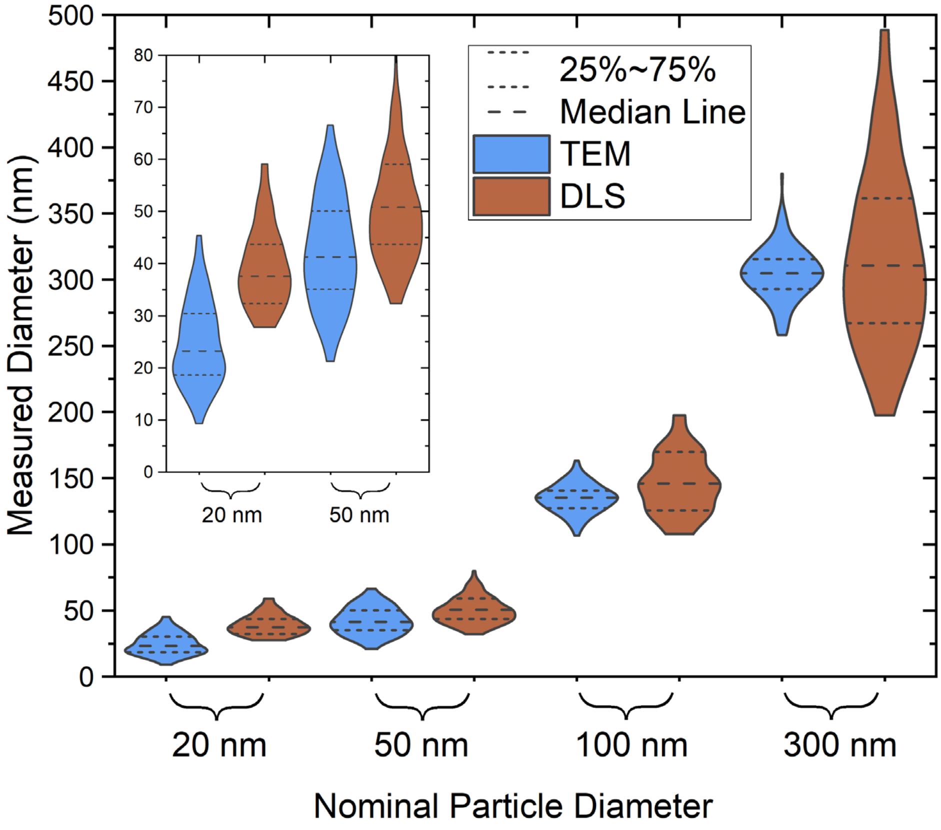

Figure S11. Size distributions for the Polystyrene NPs measured by TEM and DLS. The nominal size of the nanoparticles is shown on the x-axis. The inset plot shows a zoomed-in region of the date for the 20 nm and 50 nm NPs. Dispersions of polystyrene nanoparticles with nominal diameter (provided by the supplier): 20nm, 50nm, 100nm, 300nm were used.

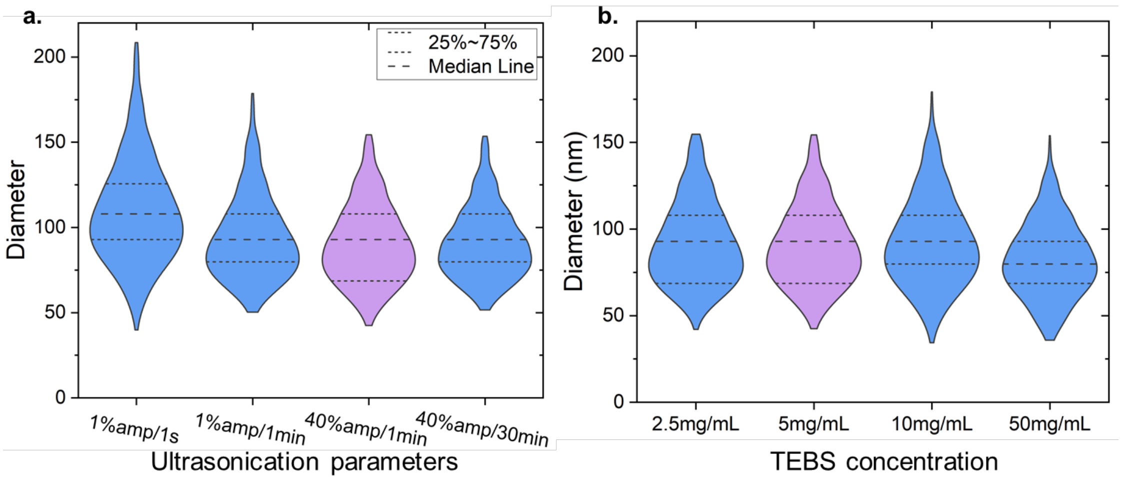

Figure S12. Size distributions for the PTB7-Th:ITIC@TEBS NPs from the aqueous phase (without evaporation of $CHCl_3$) measured by DLS. The NPs were formed with: **a)** different time and power of ultrasonication (percent amplitude) and **b)** different TEBS concentration. The purple distribution represents the same "standard" sample in both the panels (also the same as in Figure S10).

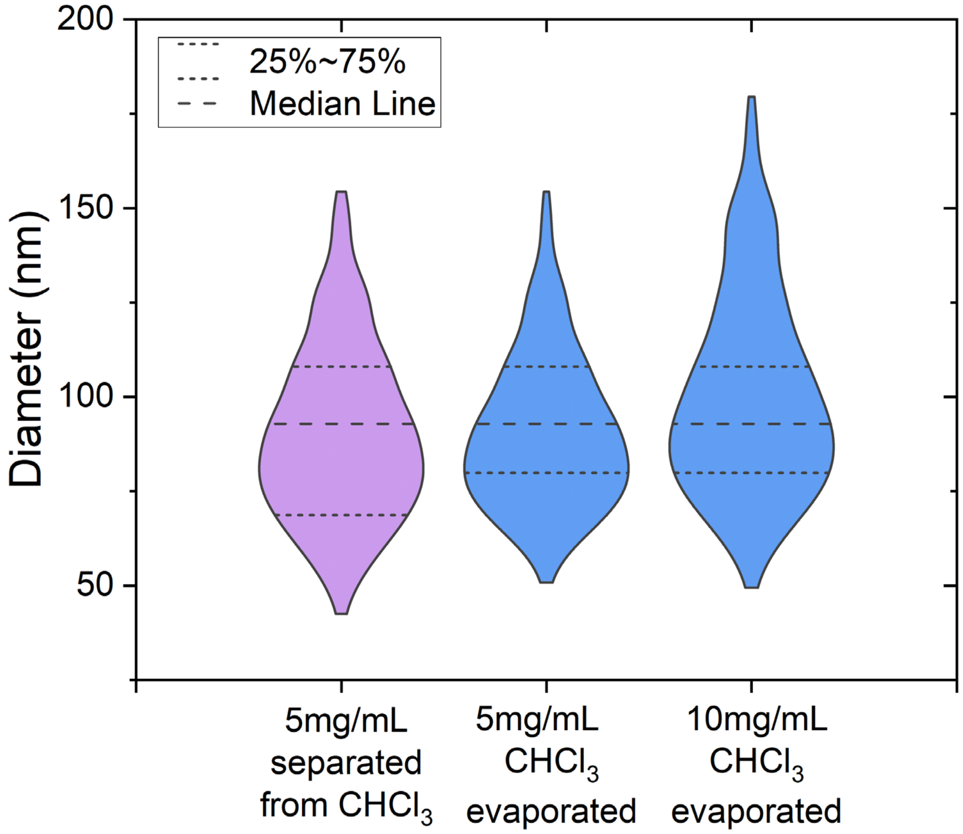

Figure S13. Size distributions for the PTB7-Th:ITIC@TEBS NPs from the aqueous phase measured by DLS and after evaporating $CHCl_3$ for different OSC concentration (blue) or

separating it by sedimentation (purple). The purple distribution represents the "standard" sample (see Figures S10 and S12 above)

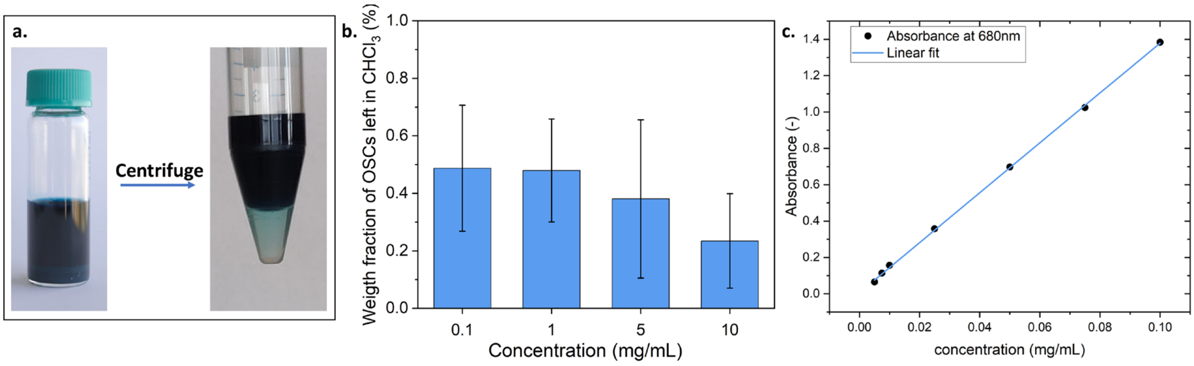

Figure S14. **a)** PTB7-Th:ITIC(3:7)@TEBS sample after ultrasonication (1min, 40amp, PTB7-Th:ITIC 5mg/mL in $CHCl_3$, TEBS 5mg/mL in water) and sedimentation of the $CHCl_3$, before (left) and after (right) centrifugation. **b)** Weight fraction of PTB7-Th:ITIC remaining in the $CHCl_3$ phase after ultrasonication, measured by UV-vis spectroscopy after centrifugation. Three samples were measured for each concentration. **c)** UV-Vis calibration curve used to quantify the amount of PTB7-Th:ITIC left in the $CHCl_3$ phase after ultrasonication.

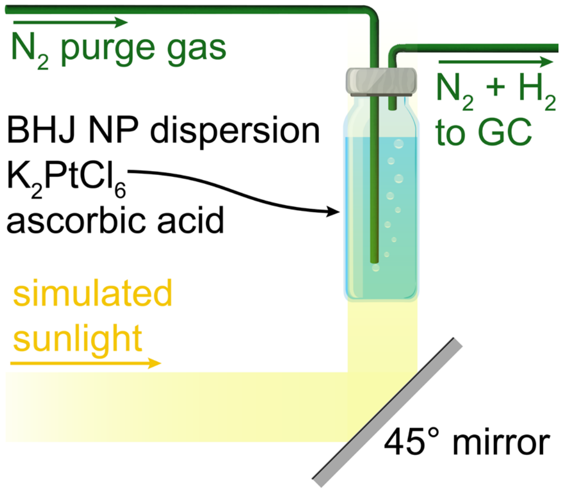

Figure S15. Schematic of the purge gas configuration used to measure the photocatalytic H_2 evolution in this work

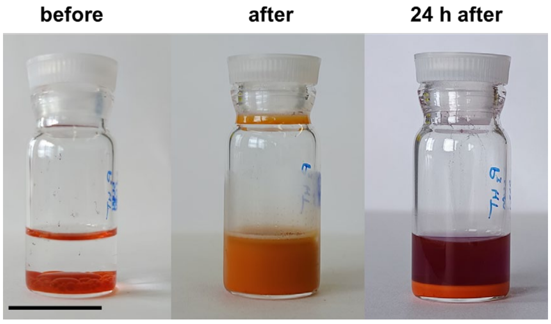

Figure S16. Photographs of the P3HT@SDS emulsion system (SDS concentration was 5 mg/mL in water) for a concentration of 5mg/mL of P3HT in $CHCl_3$ at different points in the preparation process (before, immediately after shear mixing at 3500 rpm, and 24 h after shear mixing), no heating was applied to remove the $CHCl_3$. The scale bar is 15 mm.

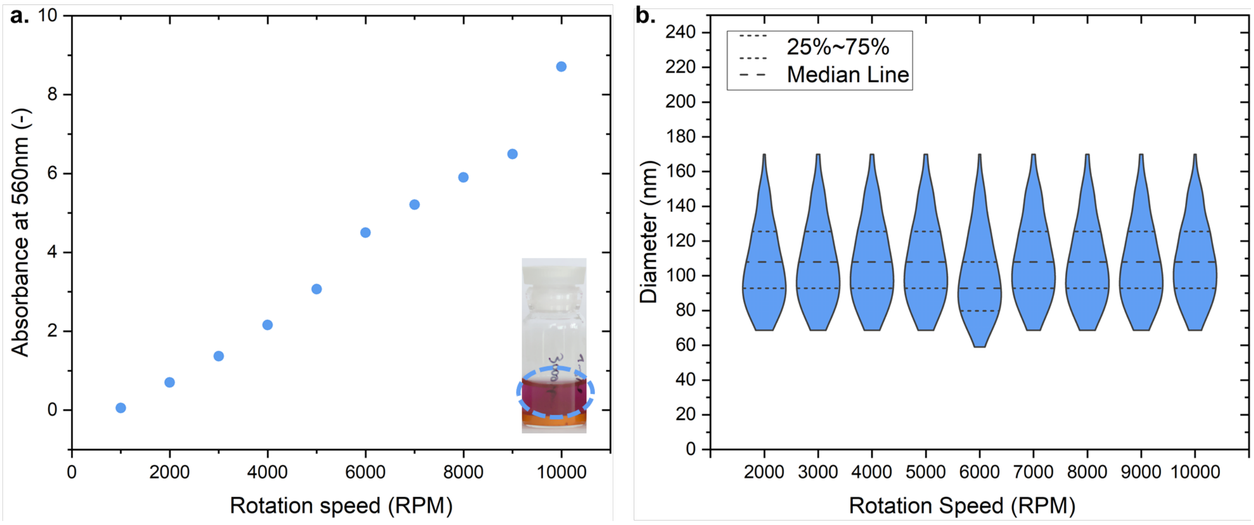

Figure S17. **a)** Absorbance of the aqueous phase after shear mixing at different rotation speed and $CHCl_3$ sedimentation for 24 h, with P3HT (1 mg/mL) and SDS (5 mg/mL). **b)** Size distributions for the P3HT@SDS NPs from the aqueous phase formed by direct extraction during shear mixing at different rotation speed measured by DLS.

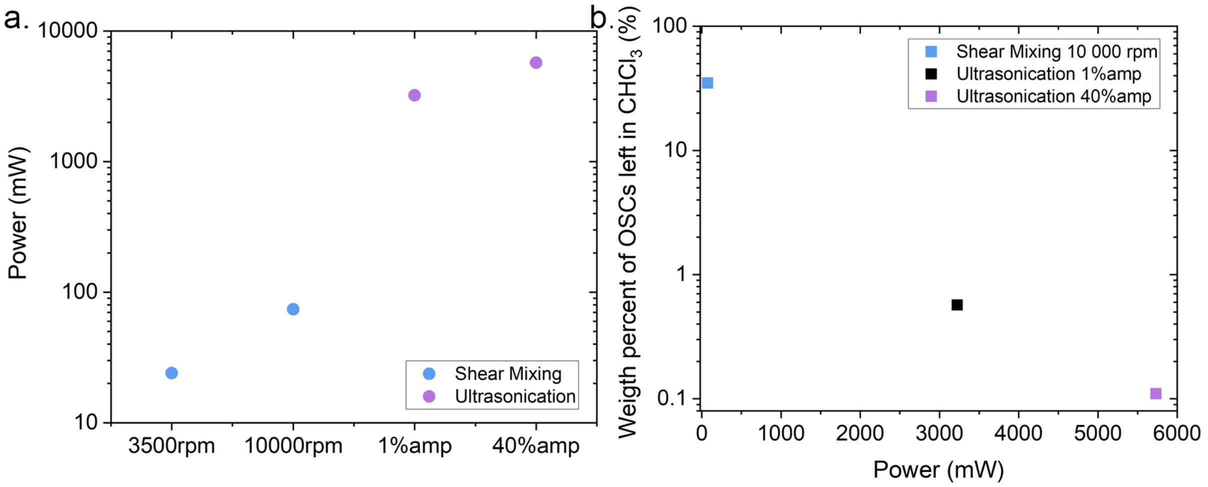

Figure S18. **a)** Measured power input during emulsification of $CHCl_3$-water-sds emulsion (SDS concentration 5mg/mL) for shear mixing at different rpm, and ultrasonication at different %amp. **b)** Weight fraction of PTB7-Th:ITIC remaining in the $CHCl_3$ phase after ultrasonication or shear mixing for the different power input (PTB7-Th:ITIC 5mg/mL in $CHCl_3$, TEBS 5mg/mL in water), measured by UV-vis spectroscopy after centrifugation.

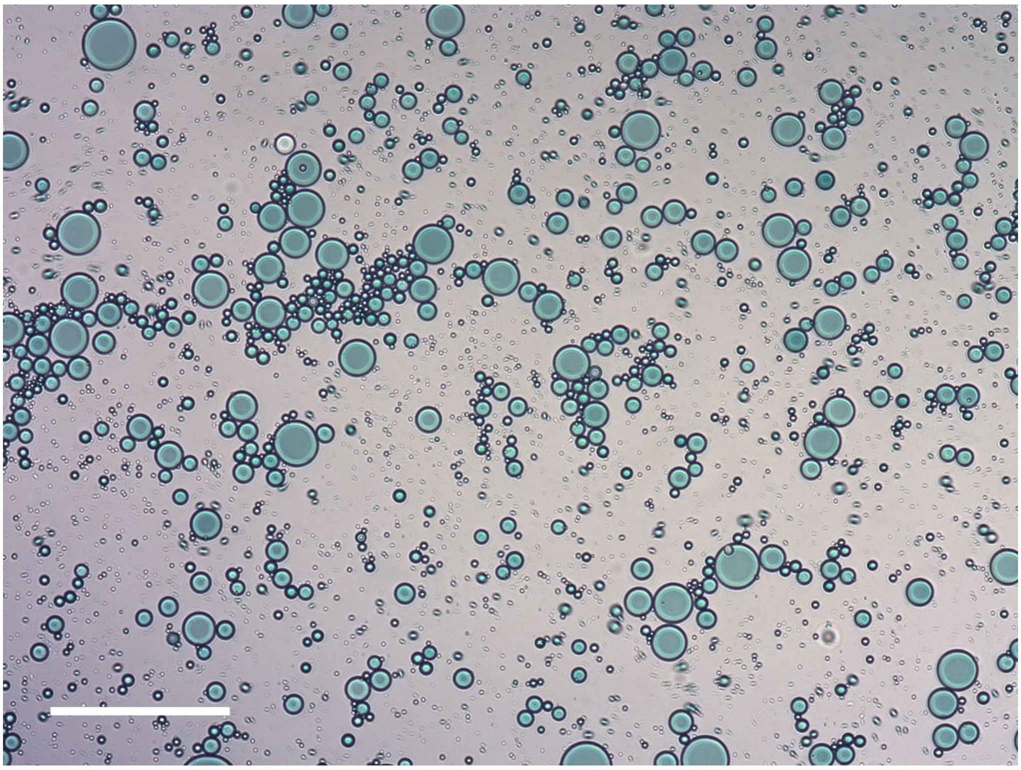

Figure S19. Optical microscope image of the emulsion obtained after shear mixing at 3500 rpm for 5 min, for the PTB7-Th:ITIC@TEBS:SDS system, with an OSC concentration of 5 mg/mL and a total surfactant concentration of 5 mg/mL. The scale bar is 100µm.

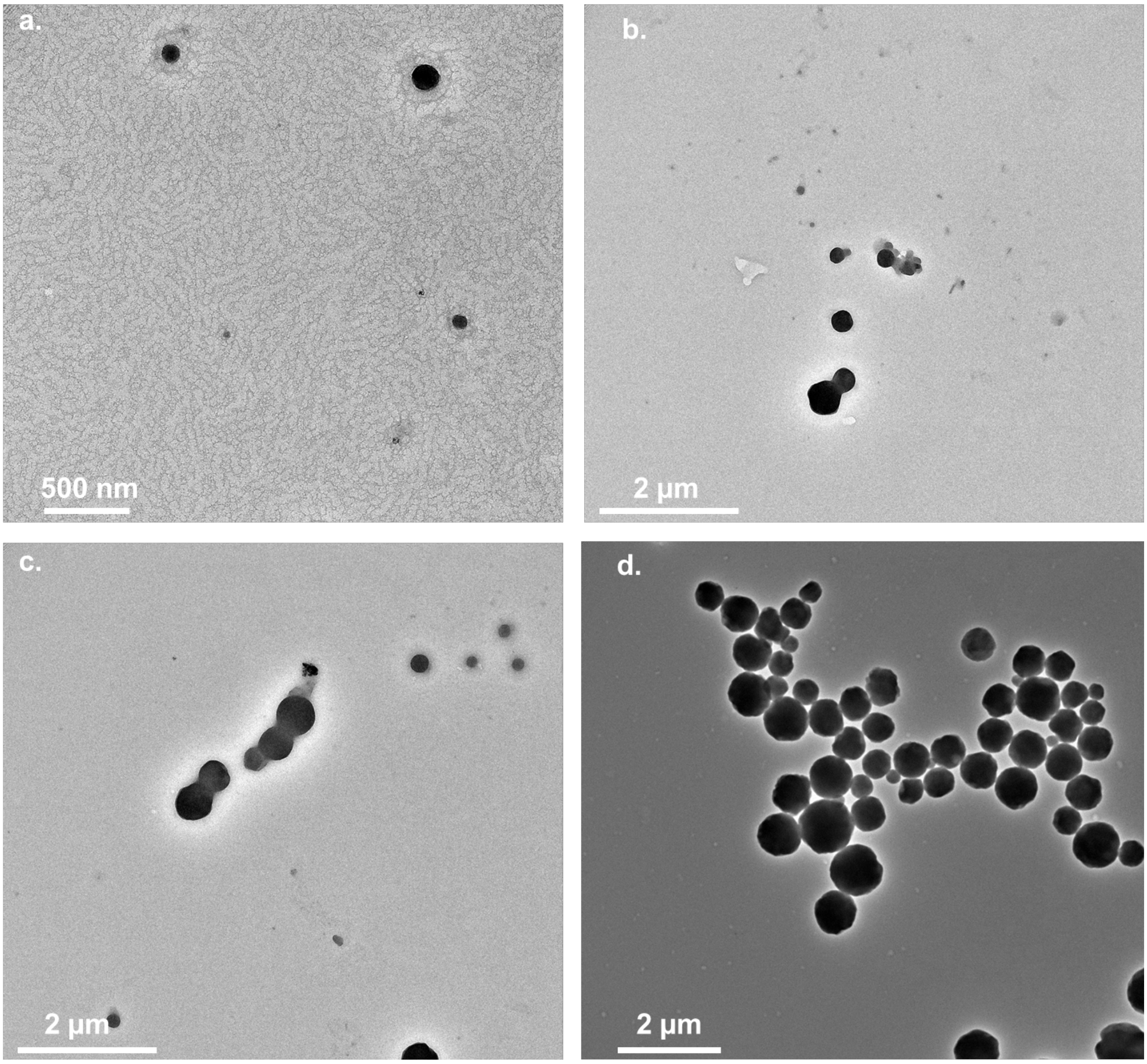

Figure S20. TEM images of the PTB7-Th:ITIC@TEBS:SDS produced via the emulsion-evaporation mechanism from shear mixing. The OSC concentrations were: **a)** 0.1 mg/mL; **b)** 0.5 mg/mL; **c)** 1 mg/mL; **d)** 5 mg/mL. The total surfactant concentration was 5 mg/mL in all cases.

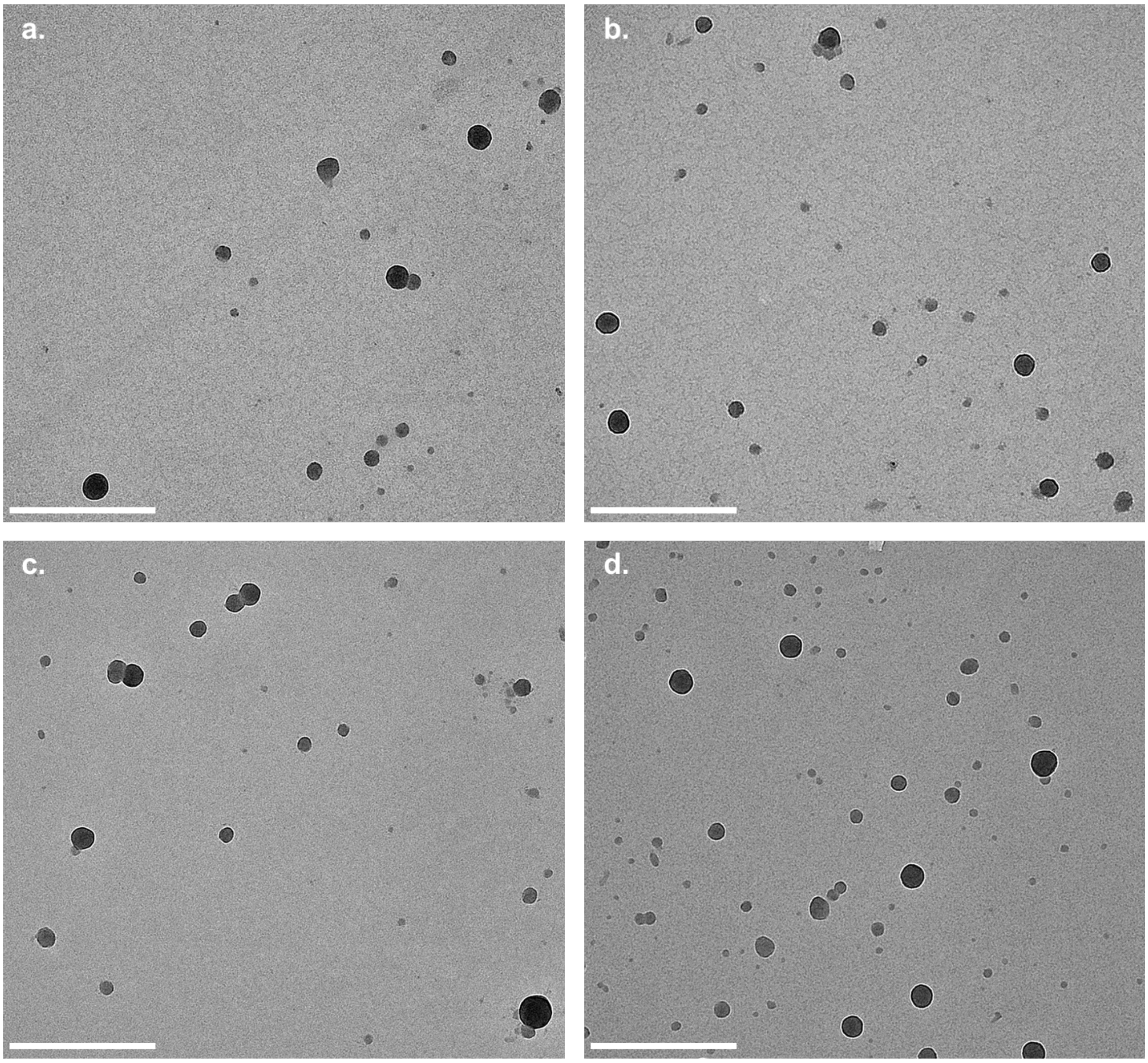

Figure S21. TEM images of PTB7-Th:ITIC@TEBS:SDS NPs formed via direct extraction during shear mixing. The OSC concentrations were: **a)** 0.1 mg/mL; **b)** 0.5 mg/mL; **c)** 1 mg/mL; **d)** 5 mg/mL. The total surfactant concentration was 5 mg/mL in all cases. The scale bars are 500nm.

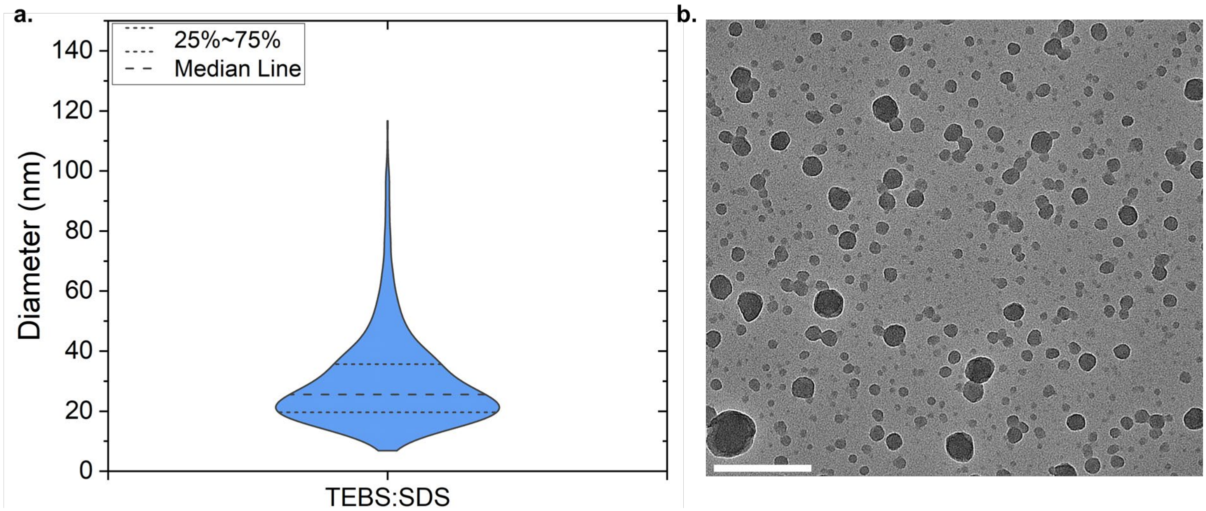

Figure S22. PTB7:ITIC@TEBS:SDS (TEBS:SDS 3:1) nanoparticles formed by ultrasonication. **a)** Size distributions from TEM. **b)** Representative TEM image. The scale bar is 200 nm.

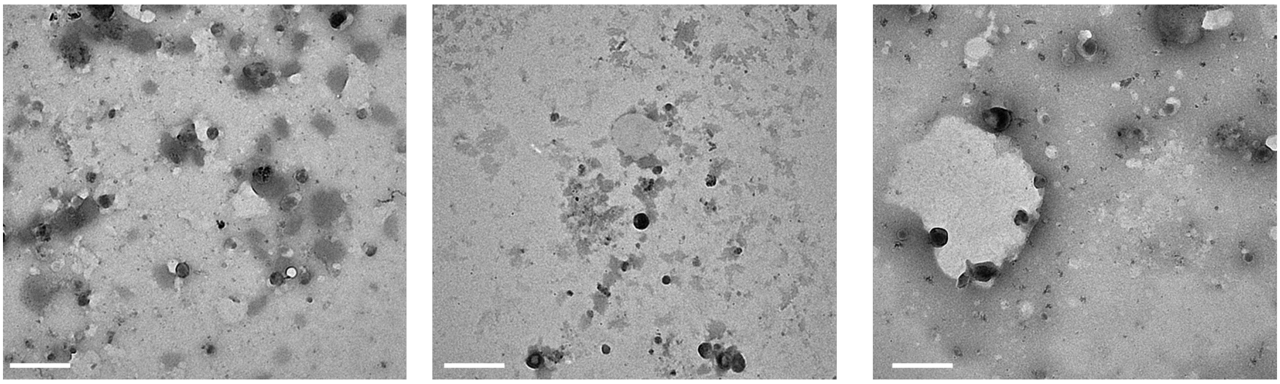

Figure S23. TEM images of the NPs produced by shear mixing with an OSC precursor concentration of 5 μg/mL, after concentrated the solution and dialyzed for 8 days. The scale bars are 500 nm.
